# The Buffering Activity of Ceria toward Reactive Oxygen Species: A Density Functional Theory Perspective

**DOI:** 10.1021/acs.jpcc.5c03050

**Published:** 2025-06-18

**Authors:** Khoa Minh Ta, Craig J. Neal, Melanie Coathup, Sudipta Seal, Lisa J. Gillie, David J. Cooke, Stephen C. Parker, Marco Molinari

**Affiliations:** † Department of Physical and Life Sciences, School of Applied Sciences, 4013 University of Huddersfield, Huddersfield HD1 3DH, U.K.; ‡ Department of Materials Science and Engineering, Advanced Materials Processing and Analysis Center, Nanoscience and Technology Center, 6243 University of Central Florida, Orlando, Florida 32816, United States; § College of Medicine, University of Central Florida, Orlando, Florida 32827, United States; ∥ Biionix Cluster, Department of Medicine, College of Medicine, University of Central Florida, Orlando, Florida 32827, United States; ⊥ Department of Chemistry, 1555 University of Bath, Claverton Down, Bath BA2 7AY, U.K.

## Abstract

Nanocrystalline ceria exhibits nanozymatic activities, which are strongly affected by surface composition and surface Ce^3+^ concentration. Here, we use density functional theory to perform a scan of the compositional landscape of the most important {111}, {110}, and {100} ceria nanoparticle surfaces and their buffering activity toward reactive oxygen species (ROS) involved in the superoxide dismutase (SOD) and catalase (CAT) enzymatic mimetic activity of ceria. This study displays that pristine and surface sublayer oxygen-deficient surfaces can perform catalytic activities, whereas surface layer oxygen-deficient surfaces can only perform noncatalytic reactions as the oxygen vacancy is healed by ROS changing surface stoichiometry. Our findings corroborate conventional literature that higher concentrations of Ce^3+^ favor SOD, whereas Ce^4+^ favors CAT while also highlighting contributions of specific subprocess reactions. {111} surfaces perform best as fully oxidized (CAT) and fully reduced (SOD), while this is not the case for the {110} and {100} surfaces. As we follow plausible reaction mechanisms of SOD and CAT, we depict a complex situation highly dependent on the surface composition, which clearly implies that it is vital to control subprocess reactions for optimal buffering, and the desorption of products is a critical step in all reactions.

## Graphical abstract

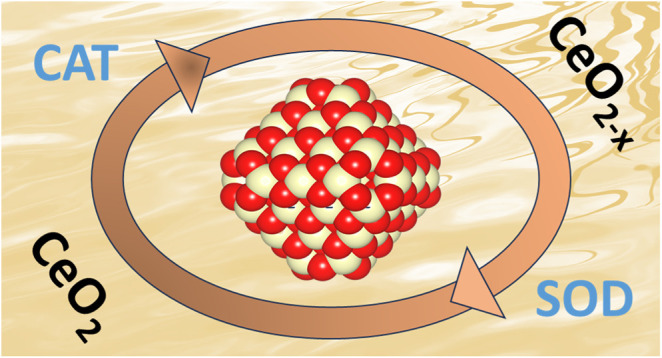

## 1 Introduction

Reactive oxygen species (ROS) are produced by normal cells’ signaling functions.^1–3^ Cells have the ability to regulate the level of ROS using antioxidant enzymes such as catalase (CAT), and superoxide dismutase (SOD).^4–6^ In excess, ROS cause human health issues, including apoptosis, necrosis inflammation, carcinogenesis, and genotoxicity.^7–9^

Nanoparticles of the rare-earth oxide, cerium oxide (CeNPs), are widely used in catalysis^10,11^ and have become promising in biomedical applications.^12–20^ CeNPs were discovered to display anti-inflammatory and antioxidant activities,^13^ preventing cytotoxicity and cell damage.^20^ These beneficial therapeutic activities are due to cerium oxide’s ability to mimic natural enzymes. This is due to the well-known oxygen storage (and release) capacity of cerium oxide, which makes CeNP formulations function as oxygen buffers that can reversibly adsorb/desorb oxygen species without significantly changing or damaging the underlying structure,^21–24^ and to its fluorite crystal structure that can accommodate cerium ions in both trivalent and tetravalent oxidation states,^21^ without structural changes.^25^ Indeed, CeNPs have been classified as nanozymes. Till now, CeNPs have been known to show SOD,^12,26,27^ CAT,^4,12,20,28,29^ phosphatase^30–34^ activities, etc.

As the size of CeNPs decreases, surface oxygen vacancies can form more easily to compensate the increase in surface energy,^35^ which would ultimately influence the Ce^3+^/Ce^4+^ ratio. Additionally, the formation energy of oxygen vacancies differs on unique surface facets (often reported to follow the trend of {111} > {100} > {110}). This gives a clear indication that the enzyme mimetic activities of CeNPs are dependent on size, morphology and surface speciation. For example, CeNPs with low Ce^3+^/Ce^4+^ ratio are found to exhibit good CAT activity, while high Ce^3+^/Ce^4+^ ratios are more beneficial for the SOD activity.^17,30^ While a growing volume of studies detailing syntheses of unique CeNP formulations with differing surface facet densities, oxygen vacancy densities, sizes, and morphologies, and utilization of such particles with purported optimized enzyme-mimetic activities have been published; observed material performances are neither yet fully explained nor represented by existing theoretical models.

The oxygen vacancies have been proposed to be fundamental for mediation of the antioxidant behavior of CeNPs.^26^ However, there is a debate in the literature whether subsurface oxygen vacancies^36–39^ or surface oxygen vacancies^35,40,41^ are the most effective toward nanozymatic activities. Unlike the stoichiometric surfaces that appear to promote the CAT activity instead of the SOD activity, oxygen deficient surfaces can perform both SOD and CAT activities, although favoring SOD, both via catalytic and noncatalytic pathways, which are determined by the location, distribution, and concentration of oxygen vacancies at the surface.^39^ Unlike in the catalytic pathway where the surface composition is unchanged after reaction, in the noncatalytic pathway, an oxygen vacancy is healed by the oxygen of the adsorbate and the surface composition is irreversibly altered.

Despite the known enzyme-mimetic activities, their underlying mechanisms of reaction are still not fully understood because of the complexity of CeNPs “defect characters”. To complicate the matter, CeNPs may express competing enzymatic mimetic activities simultaneously, all controlled by different factors, such as morphology and surface composition (e.g., stoichiometry, Ce^3+^/Ce^4+^ ratio). Furthermore, the presence of surface species, such as residues from synthesis conditions or material application environments, can show unique preferences for chemistry-relevant defect structures (e.g., Ce^3+^ sites, vacancies, specific facets) and can influence surface chemistry.^42–45^ For instance, adsorption of cerium precursor counterions (e.g., chlorides) and biological phosphates have been shown to modulate SOD and/or CAT activities.^29,43,46–48^ Modulation is suggested to occur due to adsorbate occupation of catalytic sites and/or exchange for hydroxyl species.^42,43,45^ Such complexity makes attribution of nanozyme performance to discrete/definable material properties more difficult, descriptions of these relationships more opaque, and effectively undermines the applicability of the CeNPs as nanozymes.

Here, we investigate the SOD and CAT activities on the three most stable {100}, {110} and {111} surfaces of ceria at different surface Ce^3+^ concentration and compositions, including pristine and oxygen deficient surfaces with surface layer and sublayer oxgyen vacancies.

## 2 Computational Methodology

Density functional theory (DFT) was implemented using the Vienna Ab initio Simulation Package (VASP).^49–51^ All calculations were carried out using the generalized gradient approximation (GGA) exchange-correlation functional of Perdew, Burke and Ernzerhof (PBE), and a plane wave cutoff energy of 500 eV with the projector augmented wave pseudopotential. The on-site Coulombic interaction of electron localization on the 4f orbital of Ce atoms is accounted for by the *U* correction of Dudarev,^52^ with *U*_eff_ = 5 eV.

The bulk full unit cell containing 4 CeO_2_ units was minimized at constant pressure with the electronic and ionic convergence criteria of 1 × 10^–5^ eV and 1 × 10^–3^ eV Å^–1^, respectively. The *k*-point grid 5 × 5 × 5 was used to sample the Brillouin zone. The minimized CeO_2_ bulk structure retains the *Fm*3̅*m* space group with a lattice constant of 5.498 Å,^53^ which is a known overestimation compared to the experimental value of 5.411 Å.^54^ The {100}, {110}, and {111} surfaces were generated using METADISE code^55^ from the minimized bulk CeO_2_ unit cell. The CeO_2_ surfaces were obtained following the slab method, which allows the top and the bottom layer of the material to relax. The {100} surface was a √2 × √2 expansion with 7 surface layers resulting in 28 CeO_2_ units. The {110} surface was a √2 × 2 expansion with 7 surface layers resulting in 28 CeO_2_ units. The {111} surface was generated as a √2 × √2 expansion with 5 surface layers resulting in 20 CeO_2_ units. The surfaces were geometrically optimized with the electronic and ionic convergence criteria of 1 × 10^–5^ eV and 1 × 10^–2^ eV Å^–1^, respectively. The *k*-point grid for surface configurations was 2 × 2 × 1 to sample the Brillouin zone. The surface energies of the {100}, {110}, and {111} surfaces are calculated to be 1.45, 1.07, and 0.70 Jm^2–^ respectively, with their order of stability following {111} > {110} > {100}, where the {111} surface is the most stable.^56^ The formation energy of an oxygen vacancy on the surface layer follows the order {111} (2.15 eV) > {100} (1.74 eV) > {110} (1.50 eV), where the {111} surface required the highest energy to remove a surface oxygen ion.^57^

All adsorbed species on the different surface configurations are labeled as *n*h-CeO_2_ (pristine surfaces), *n*h-CeO_2–*x*(ssl)_ (surface sublayer oxygen deficient surfaces) and *n*h-CeO_2–*x*(sl)_ (surface layer oxygen deficient surfaces), where *n* is the number of hydrogen atoms chemisorbed on the surface oxygen atoms of the surface. This allows us to adjust the Ce^3+^ concentration as each chemisorbed H atom introduces an electron into the surface reducing one surface Ce^4+^ ion to Ce^3+^; hence one Ce^3+^ is introduced for every H atom chemisorbed. For example, when *n* = 2, there are 2 H atoms chemisorbed on the surface of CeO_2_ and the label is 2h-CeO_2_, resulting in a dihydroxylated pristine surface. Furthermore, the difference between pristine and oxygen deficient surfaces is the presence of an oxygen vacancy, which introduces 2 × Ce^3+^. Thus, the difference in terms of surface Ce^3+^ concentration between the pristine and the oxygen deficient surfaces is always at least 2 × Ce^3+^. Here, we refer to the surface fractional coverage of Ce^3+^, *x*_s_(Ce^3+^), of 0, 0.25, 0.5, 0.75 and 1, which correspond to 0, 1, 2, 3, and 4 surface Ce^3+^. As our configurations have 4 surface Ce ions, we have studied from fully oxidized (stoichiometric) surfaces to fully reduced surfaces, which is representative of a large variety of nanoparticle compositions.

All adsorbed species were positioned to maximize interaction with the surface in terms of direct bonds with surface Ce ions and, where available, the hydrogen bond network. This approximation has been used in previous studies.^39,58,59^ Only the most stable surface configurations are represented and discussed here.

All reaction energies for all the steps included in the mechanisms of reaction are calculated as

1 $$E_{react}=\frac{\sum E_{products}-\sum E_{reac\,\tan ts}}{2}$$

where *E*_products_ and *E*_reactants_ are the energies of the product and the reactants, while the factor 2 is essential as we use the slab method where the adsorbates are present on the top and bottom of the slab in an identical manner. The reported energies for the reaction pathways are not activation energies. Whereas the description of a transition states is important, the complexity of defining and calculating energies along the reaction coordinates for all the steps is a considerable challenge and would usefully be a focus of future work. Perhaps more efficiently, experimental detection of intermediates would offer valuable support for the proposed mechanism.

## 3 Results and Discussion

Here, we present the catalytic (Section 3.1) and noncatalytic pathways (Section 3.2), where the surface composition is unaltered after reaction in the former, while an oxygen vacancy is healed by the oxygen of the adsorbate and the surface composition is irreversibly altered in the latter.

### 3.1 Catalytic Pathways

#### 3.1.1 The Superoxide Dismutase (SOD) Activity of Ceria

The superoxide dismutase activity is the conversion of the superoxide radicals HO_2_^•^ (HOO^•^), an aqueous form of the superoxide anion radicals O_2_^•–^ (OO^•–^), into hydrogen peroxide (H_2_O_2_) and oxygen (O_2_) according to eq 2

2 $$2{\mathrm{HO}}_{2}^{•}\to H_{2}O_{2}+O_{2}$$

Considering eq 2, we propose catalytic mechanisms of SOD reaction on ceria surfaces without oxygen vacancies (referred to as pristine surfaces), and oxygen-deficient ceria surfaces with oxygen vacancies positioned in the surface sublayer (referred to as ssl oxygen deficient surfaces). Oxygen vacancies can also be positioned on the surface layer, but such models allow for the healing of the vacancy resulting in noncatalytic reaction mechanisms (discussed in Section 3.2.1), which do not allow for the reaction in eq 2. We label the 7 mechanisms identified in this work as MX, where M stands for mechanism and X is a number, as shown in Table 1. All mechanisms are depicted schematically in Figure 1.

**Figure 1:**
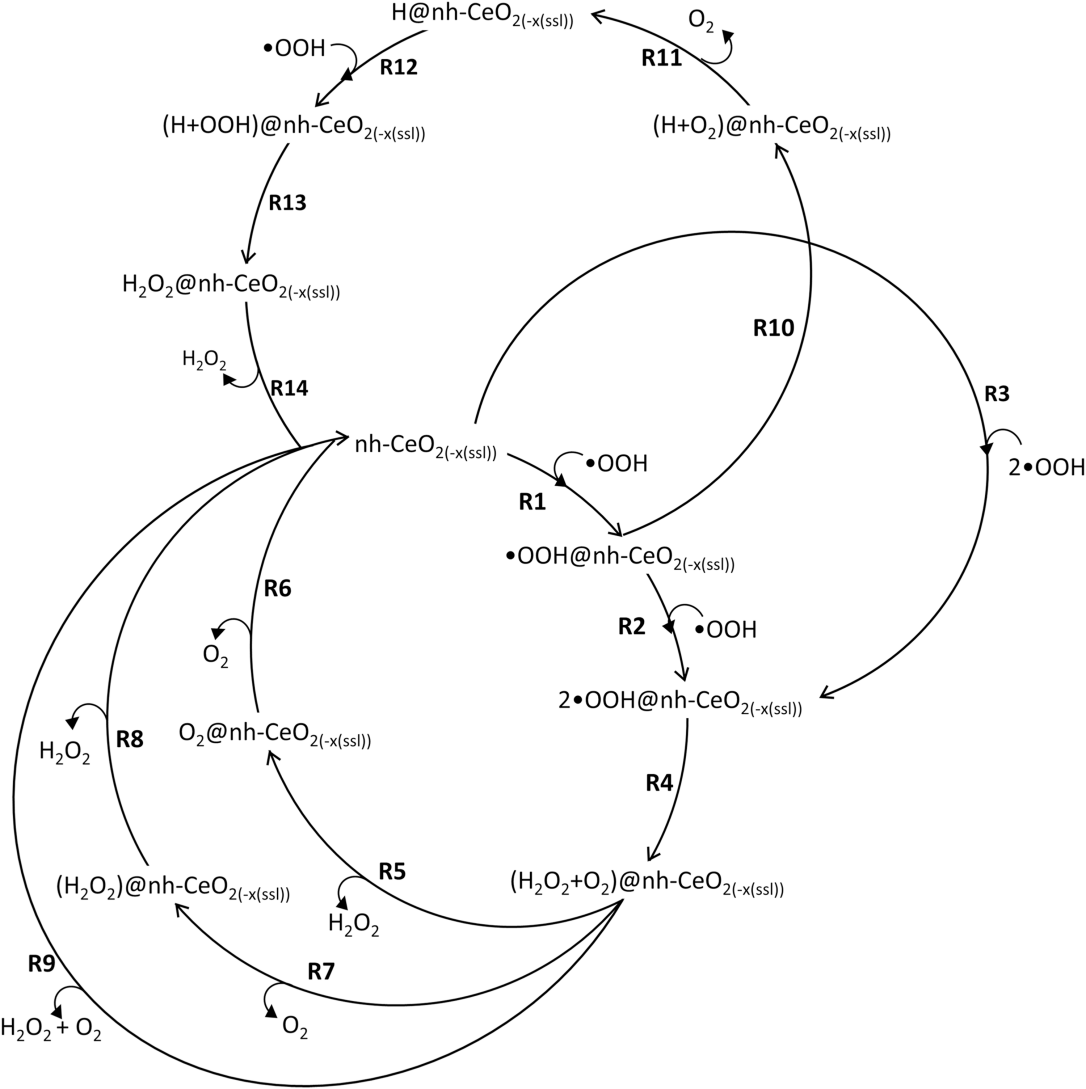
SOD catalytic reaction schemes, applied to the {100}, {110}, and {111} ceria surfaces. The reaction steps are defined by R*y*, where *y* is a number between 1 and 14. The notation *n*h-CeO_2–*x*(ssl)_ represents both pristine (*n*h-CeO_2_) and surface sublayer oxygen deficient (*n*h-CeO_2–*x*(ssl)_) ceria. In sublayer oxygen deficient (*n*h-CeO_2–*x*(ssl)_) ceria, the presence of an oxygen vacancy introduces 2 Ce^3+^ ions into the surface. *n*h represents the number (*n*) of hydroxyl groups at the surface, with *n* varying from 0 to 2, with each introducing 1 Ce^3+^ into the surface.

**Table 1:** Catalytic Reaction Mechanism of SOD According to Figure 1

mechanism	reaction included
M1	R1 + R2 + R4 + R5 + R6
M2	R1 + R2 + R4 + R7 + R8
M3	R1 + R2 + R4 + R9
M4	R3 + R4 + R5 + R6
M5	R3 + R4 + R7 + R8
M6	R3 + R4 + R9
M7	R1 + R10 + R11 + R12 + R13 + R14

Two HOO^•^ can adsorb consecutively (R1, R2) or simultaneously (R3). Either way, two HOO^•^ are adsorbed at the surface of nanoceria. Here, they convert into O_2_ and H_2_O_2_ (R4). H_2_O_2_ can then desorb first (R5) followed by O_2_ (R6), or vice versa, O_2_ first (R7) followed by H_2_O_2_ (R8), or the two molecules could desorb together (R9) to give back the starting surface. The first HOO^•^ could also adsorb (R1) and then convert into O_2_ leaving a chemisorbed H atom on a surface oxygen (R10). The second HOO^•^ could adsorb (R12), after O_2_ desorption (R11), to form H_2_O_2_ (R13) adsorbed at the surface on nanoceria, which then desorbs (R14) to leave the starting surface.

Hereafter we discuss the mechanisms in groups that have similar pathways and steps.

##### 3.1.1.1 Mechanisms M1, M2, and M3

The mechanisms M1, M2, M3 are similar as HOO^•^ radicals are adsorbed consecutively (R1 and R2), followed by their conversion into H_2_O_2_ and O_2_ within a single step (R4). The difference between M1, M2 and M3 lies in the route by which H_2_O_2_ and O_2_ desorption occurs (Figure 1), where M1 sees H_2_O_2_ desorption first (R5) followed by O_2_ (R6), M2 sees O_2_ desorption first (R7) followed by H_2_O_2_ (R8), and M3 sees both products desorbing together (R9). As we have studied different surface compositions for each mechanism, including bare and hydroxylated, pristine and sublayer oxygen deficient surfaces, we present the complete energetics for M1 and M2 mechanisms (Figure 2). In M3, the simultaneous desorption of H_2_O_2_ and O_2_ (R9) requires a high energy of desorption, making it a less viable pathway for the SOD reaction. Hence, the complete energetics for M3 is shown in Figure S4.

**Figure 2:**
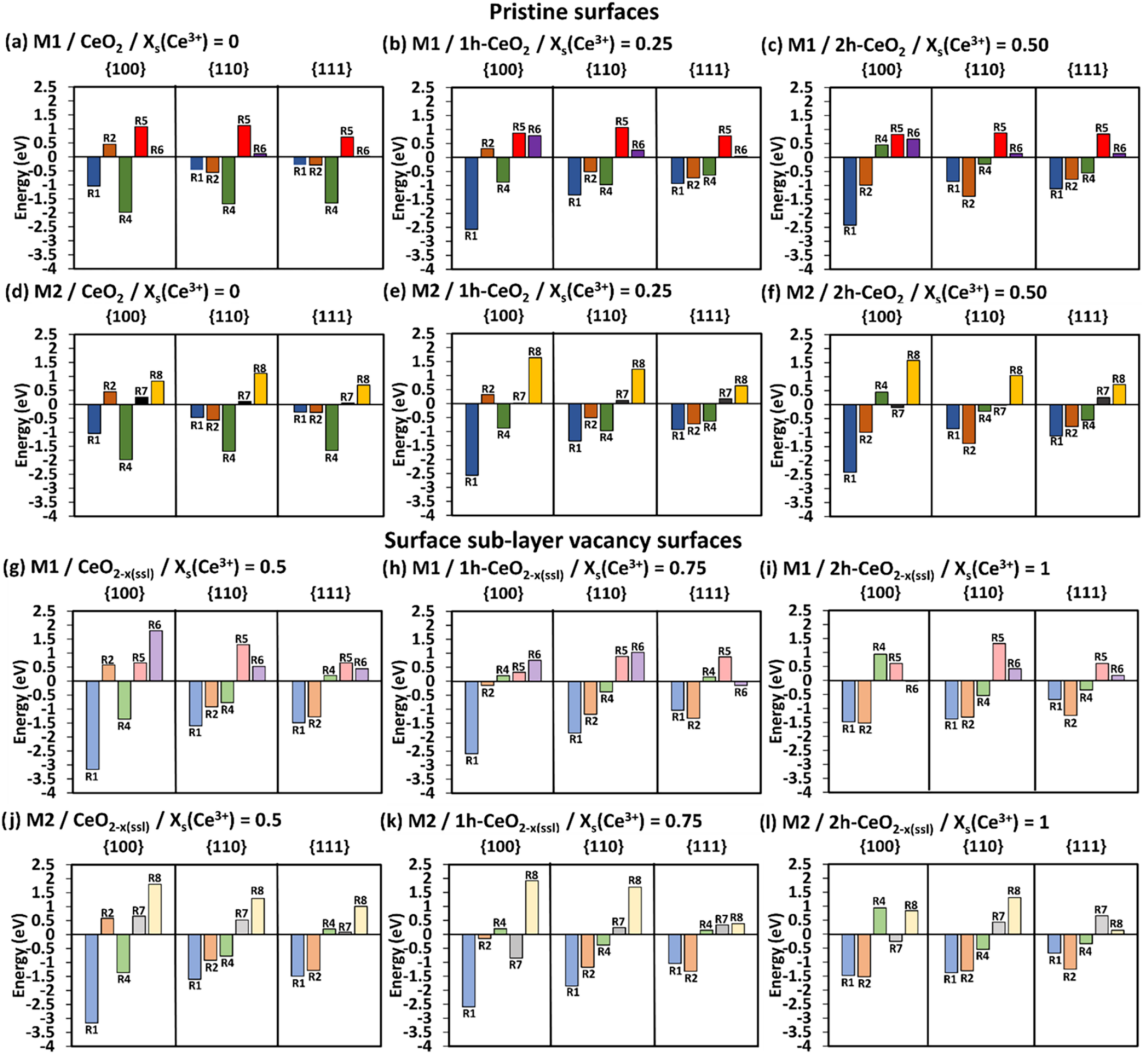
SOD catalytic activity. The complete energetics following mechanisms M1 (a–c) and M2 (d–f) on pristine surfaces and M1 (g–i) and M2 (j–l) on surface sublayer oxygen deficient surfaces at different *x*_s_(Ce^3+^). The darker colored bars are for the pristine surfaces, and the lighter color bars are for the surface sublayer oxygen deficient surfaces. R1/R2 = adsorption of HOO^•^ radicals, R4 = formation of H_2_O_2_ and O_2_. R5/R8 = desorption of H_2_O_2_, R6/R7 = desorption of O_2_.

All mechanisms see the consecutive adsorption of HOO^•^ radicals (R1 and R2), but the adsorption of the second radical (R2) is generally less favorable than the adsorption of the first radical (R1), Figure 2a–l, most likely due to the high electron density of the HOO^•^ radicals, which repel each other.^60^ However, unlike the {110} and {111} surfaces, the {100} surface shows some positive adsorption energies for the second HOO^•^ radical (R2) (Figure 2a,b pristine surfaces M1, Figure 2d,e pristine surfaces M2, Figure 2g oxygen deficient M1, and Figure 2j oxygen deficient M2). For both M1 and M2, it is clear that R2 for the {100} becomes favorable as *x*_s_(Ce^3+^) increases on both the pristine (from *x*_s_(Ce^3+^) = 0.5), and oxygen deficient (from *x*_s_(Ce^3+^) = 0.75) surfaces. We noticed that as the surface fractional coverage of Ce^3+^ increases, the second HOO^•^ adsorption (R2) becomes more favorable for all of the pristine surfaces (Figure 2a–f) and oxygen deficient surfaces (Figure 2g–l) for both M1 and M2. This is not the case for the first HOO^•^ adsorption (R1), where, as *x*_s_(Ce^3+^) increases, it becomes more favorable on pristine surfaces (Figure 2a–f) but less favorable for oxygen deficient surfaces (Figure 2g–l) for both M1 and M2. In general, this suggests that the introduction of Ce^3+^ benefits the HOO^•^ radical adsorption, i.e., Ce^3+^ acts as a stronger binding site compared to Ce^4+^. Indeed, the localized electron on the 4f orbital of the Ce^3+^ ion quenches the radical resulting in more stable adsorbed species, i.e., Ce^4+^ ion and hydroperoxide anion (HOO^–^).

The conversion of two HOO^•^ into H_2_O_2_ and O_2_ (R4) is generally spontaneous on the pristine surfaces (Figure 2a–f), apart from the {100} pristine surface for both M1 and M2, where the adsorption energies are positive and unfavorable for *x*_s_(Ce^3+^) = 0.5. Indeed, for all pristine surfaces (Figure 2a–f), R4 becomes less favorable as *x*_s_(Ce^3+^) increases. This applies also for the {100} and {110} oxygen deficient surfaces for both M1 and M2 (Figure 2g–l), however, here R4 becomes unfavorable on the {100} surface at *x*_s_(Ce^3+^) = 0.75 (Figure 2h,k,i,l). In contrast, the increase of the *x*_s_(Ce^3+^) helps the conversion step R4 on the oxygen deficient {111} surface for both M1 and M2; indeed it is unfavorable for *x*_s_(Ce^3+^) up to 0.75 (Figure 2g,h,j,k), and then it becomes favorable for the *x*_s_(Ce^3+^) = 1 (Figure 2i,l).

The last step of each mechanism is the desorption of O_2_ and H_2_O_2_, which is generally unfavorable, Figure 2 for M1 and M2, and Figure S4 for M3. The only exceptions of negative desorption energies are on the oxygen deficient {111} surface (M1 R6 = −0.15 eV in Figure 2h), and on the {100} surface (M1 R6 = −0.03 eV in Figure 2i, M2 R7 = −0.85 eV in Figure 2k, and M2 R7= −0.26 eV in Figure 2l). The departure of H_2_O_2_ and O_2_ molecules from the surface would provide available space for the next HOO^•^ radicals to adsorb and to continue the catalytic cycle; hence, lower positive desorption energies for H_2_O_2_ and O_2_ would be more beneficial for the SOD catalytic reaction to continue. Generally, the desorption of H_2_O_2_ (M1 R5 and M2 R8) is less favorable (i.e., higher positive energies) than that of O_2_ (M1 R6 and M2 R7) for both M1 and M2 on both pristine and oxygen deficient surfaces. Looking at the desorption of H_2_O_2_ from pristine surfaces, it is comparably more favorable on the {111} followed by the {100} and {110} surfaces for M1, but by {110} and {100} surfaces for M2. The same behavior is generally seen on the oxygen deficient surfaces for M2 (apart from *x*_s_(Ce^3+^) = 1, Figure 2l, where H_2_O_2_ desorption follows {111} then {100} and {110} with {111} the most favorable), although M1 does not show a clear trend but still has the {111} surface displaying the lowest energy. The desorption of H_2_O_2_ from pristine surfaces for M1 shows relatively stable energies across all *x*_s_(Ce^3+^) (Figure 2a–c), whereas the *x*_s_(Ce^3+^) = 0 for M2 shows the lowest energies of desorption (R8 {100} = 0.83 eV, R8 {110} = 1.11 eV, and R8 {111} = 0.69 eV, Figure 2d). For oxygen deficient surfaces, desorption of H_2_O_2_ has lower energies for higher *x*_s_(Ce^3+^) = 1 for M2 and again does not show any specific trend for M1. Hence, our data implies that the expression of {111} surfaces would be more beneficial for the SOD activity as products desorb more easily leaving surface binding sites available to restart the SOD reaction again. Indeed, most of the SOD catalysis on nanoceria is performed on {111} surfaces as many experiments have shown that truncated octahedral and octahedral shapes are best in terms of SOD performance.^61–63^

In general, a nanozyme with good SOD activity would be able to adsorb as many HOO^•^ radicals to start the cycle. Our data in Figure 2 shows that most of our surfaces and compositions would be able to do this, indicating that R1 and R2 are not limiting reaction steps. The same applies to the conversion of the radicals to H_2_O_2_ and O_2_ (R4), which again is largely favorable. Although it is complicated to pinpoint the best surfaces and compositions, one could imagine that those surfaces and compositions with the lowest conversion R4 would be the most suitable for the SOD activity, of course as far as the adsorption of the HOO^•^ radicals are also favorable. Finally, and as important as the conversion R4 is, the desorption of the reaction products, H_2_O_2_ and O_2_, would also be important as surface sites will need to be freed to start the catalytic cycle from the start. So perhaps we can conclude that there are three important points:

(1) the adsorption of the HOO^•^ radicals (R1 and R2) should be favorable
(2) the conversion of HOO^•^ radicals into H_2_O_2_ and O_2_ (R4) should be as favorable as possible
(3) the desorption of H_2_O_2_ and O_2_ should be as favorable as possible. Here the energies are generally positive and the desorption of H_2_O_2_ appears to be the one with the highest energy, so lower energies of desorption of H_2_O_2_ would be preferred.

Considering the three criteria, the best compromise for SOD activity would be M1 oxygen-deficient {100} and {110} surfaces with *x*_s_(Ce^3+^) = 0.75, and M2 oxygen-deficient {111} surface with *x*_s_(Ce^3+^) = 1.

##### 3.1.1.2 Mechanisms M4, M5, and M6

The mechanisms M4, M5 and M6 are similar to the mechanisms M1, M2 and M3, except that the consecutive adsorption of HOO^•^ radicals (R1, R2) in M1, M2, and M3 is replaced by the simultaneous adsorption of two HOO^•^ radicals (R3) in M4, M5 and M6, respectively. M4, M5 and M6 share step R3, but differ in the conversion of HOO^•^ radicals into H_2_O_2_ and O_2_ (R4), and in the desorption of H_2_O_2_ and O_2_, where M4 sees H_2_O_2_ desorption first (R5) followed by O_2_ (R6) as for M1, M5 sees O_2_ desorption first (R7) followed by H_2_O_2_ (R8) as for M2, and M6 sees both products desorbing together (R9) as for M3. The complete energetics of M4 is shown in Figure 3 as an example, and in Figures S5–S6 for M5 and M6.

**Figure 3:**
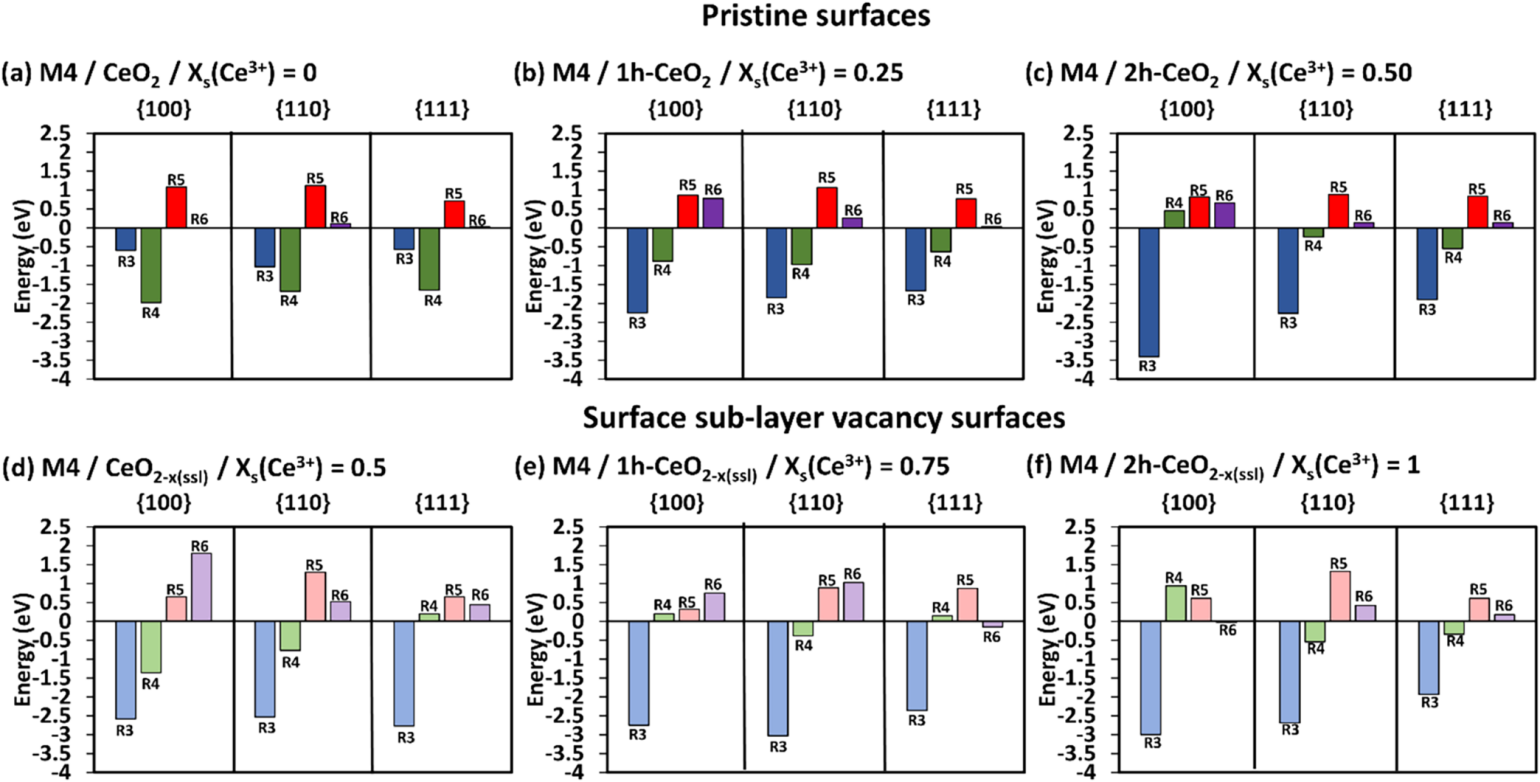
SOD catalytic activity. The complete energetics following mechanisms M4 (a–c) on pristine surfaces and M4 (d–f) on surface sublayer oxygen deficient surfaces at different *x*_s_(Ce^3+^). The darker colored bars are for the pristine surfaces, and the lighter color bars are for the surface sublayer oxygen deficient surfaces. R3 = adsorption of HOO^•^ radicals, R4 = formation of H_2_O_2_ and O_2_, R5 = desorption of H_2_O_2_, R6 = desorption of O_2_.

The simultaneous adsorption of two HOO^•^ radicals (R3) is favorable (M4–M6). This gives an indication that this simultaneous adsorption is more favorable than the consecutive adsorption of two HOO^•^ radicals as in M1–M3.

The adsorption step R3 on pristine surfaces with *x*_s_(Ce^3+^) = 0 (Figure 3a) follow the trend {110} (−1.03 eV) < {100} (−0.60 eV) ≈ {111} (−0.57 eV), where the {110} surface shows the most stable HOO^•^ radical adsorption. The increase of *x*_s_(Ce^3+^) on pristine surfaces enhances the adsorption stability on all three surfaces, and it switches the order of adsorption stability to {100} < {110} < {111} at *x*_s_(Ce^3+^) = 0.25, with {100} shows the most stable adsorption.

On the oxygen deficient surfaces with *x*_s_(Ce^3+^) = 0.50, the adsorption of HOO^•^ radicals is preferred on the {111} surface (Figure 3d), followed by the {100} ≈ {110} surfaces, (i.e., {111} (−2.77 eV) < {100} (−2.58 eV) ≈ {110} (−2.53 eV)). The increase of *x*_s_(Ce^3+^) on the oxygen deficient surfaces enhances the adsorption stability of HOO^•^ radicals on the {100} and {110} surfaces but not on the {111}. The {100} (−3.00 eV) and {110} (−2.69 eV) oxygen deficient surfaces with *x*_s_(Ce^3+^) = 1 have the most negative R3 energies (Figure 3f), while the {111} (−1.93 eV) oxygen deficient surface with *x*_s_(Ce^3+^) = 0.50 shows the most negative R3 energy (Figure 3d).

Overall, the increase of *x*_s_(Ce^3+^) in the {100}, {110} and {111} pristine surfaces enhance the HOO^•^ radicals adsorption stability. Similarly, the high *x*_s_(Ce^3+^) in the oxygen deficient surfaces promotes the HOO^•^ radical adsorption on {100} and {110} surfaces, but not on the {111} surface. The comments on the conversion of HOO^•^ radicals into H_2_O_2_ and O_2_ (R4), and the desorption of H_2_O_2_ and O_2_, (R5, R6 for M4, R7 and R8 for M5, and R9 for M6) remain the same as in Section 3.1.1 for M1–M3. Hence, the best compromise for SOD activity would be M4 oxygen-deficient {100} and {110} surfaces with *x*_s_(Ce^3+^) = 0.75, and M5 oxygen-deficient {111} surface with *x*_s_(Ce^3+^) = 1. This would also put the mechanisms M4 and M5 ahead of M1 and M2 discussed in Section 3.1.1, as the simultaneous adsorption (R3) is always more stable than the consecutive adsorption (R1 and R2) of HOO^•^ radicals.

##### 3.1.1.3 Mechanism M7

For M7, a HOO^•^ radical is adsorbed (R1), followed by the conversion into O_2_ (R10) and O_2_ desorption (R11), after which a second HOO^•^ radical adsorbs (R12) followed by the conversion into H_2_O_2_ (R13), and then H_2_O_2_ desorption (R14). The complete energetics of mechanism M7 are shown in Figure 4.

**Figure 4:**
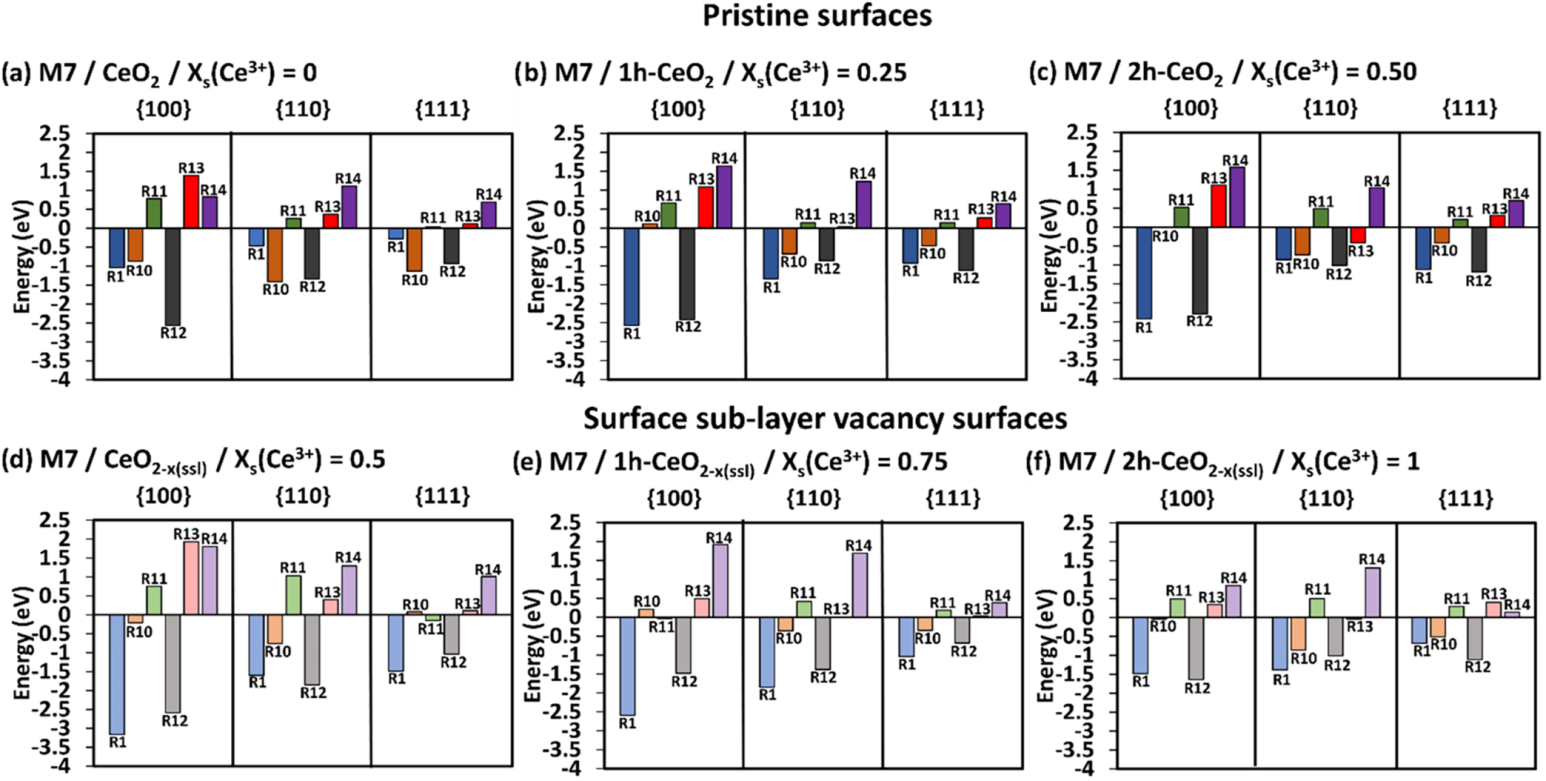
SOD catalytic activity. The complete energetics following mechanisms M7 (a–c) on pristine surfaces and M7 (d–f) on surface sublayer oxygen deficient surfaces at different *x*_s_(Ce^3+^). The darker colored bars are for the pristine surfaces, and the lighter color bars are for the surface sublayer oxygen deficient surfaces. R1/R12 = adsorption of HOO^•^, R10 = formation of O_2_, R11 = desorption of O_2_, R13 = formation of H_2_O_2_, R14 = desorption of H_2_O_2_.

The adsorption energies of the HOO^•^ radicals (R1 and R12) are favorable processes on both pristine and oxygen deficient surfaces. The increase in *x*_s_(Ce^3+^) on the pristine surfaces helps stabilize the HOO^•^ radical adsorption where both energies for R1 and R12 become favorable (more negative, Figure 4a–c). The oxygen deficient surfaces behave in the opposite manner compared to pristine surfaces, where the increase in *x*_s_(Ce^3+^) destabilizes the adsorption energies for both R1 and R12 (Figure 4d–f).

The HOO^•^ radical adsorbed first is converted into O_2_ (R10) leaving a hydrogen chemisorbed on the surface. Figure 4 shows that the formation of O_2_ is generally favorable on both pristine and oxygen deficient surfaces. There are a few exceptions where the formation of *O*_2_ shows positive energies: {100} pristine surface = 0.11 eV (Figure 4b), {100} oxygen deficient surface = 0.21 eV (Figure 4e), and {111} oxygen deficient surface = 0.08 eV (Figure 4d). The O_2_ formation on pristine surfaces (Figure 4a–c) is favored on the {110}, followed by {111} and {100} surfaces across all *x*_s_(Ce^3+^). When *x*_s_(Ce^3+^) = 0 (Figure 4a) the lowest O_2_ formation energies on pristine surfaces are displayed with the order of stability of {100} (−0.87 eV) < {111} (−1.13 eV) < {110} (−1.41 eV). A similar order of stability for the formation of O_2_ is observed on the oxygen deficient surfaces, except when *x*_s_(Ce^3+^) = 0.50 (Figure 4d), where the order follows {111} (0.08 eV) < {100} (−0.21 eV) < {110} (−0.76 eV), with the {110} surface being the most favorable facet. Unlike the oxygen deficient {100} surface, which shows the lowest O_2_ formation energy at *x*_s_(Ce^3+^) = 0.50 (R10 {100} = −0.21 eV, Figure 4d), both oxygen deficient {110} and {111} surfaces exhibit the lowest energies of O_2_ formation at *x*_s_(Ce^3+^) = 1 (R10 {110} = −0.86 eV and R10 {111} = −0.51 eV, Figure 4f).

Unlike the formation of O_2_, the conversion of the second adsorbed HOO^•^ into H_2_O_2_ (R13) is generally an unfavorable process, with two exceptions for the {110} pristine and oxygen deficient surfaces where R13 are negative ({110} pristine surface = −0.41 eV, Figure 4c, and {110} oxygen deficient surface = −0.04 eV, Figure 4f).

The H_2_O_2_ formation on both pristine and oxygen deficient {100} surfaces is generally the most unfavorable compared to the other two surfaces, except for the oxygen deficient {111} surface at *x*_s_(Ce^3+^) = 1, Figure 4f (R13 {111} = 0.4 eV vs R13 {100} = 0.34 eV). For both pristine (*x*_s_(Ce^3+^) = 0, Figure 4a) and oxygen deficient (*x*_s_(Ce^3+^) = 0.5, Figure 4d) surfaces, the formation of H_2_O_2_ (R13) is less energetically demanding on the {111}, followed by {110} and then {100} surfaces. However, an increase in *x*_s_(Ce^3+^) on pristine surfaces changes the order of H_2_O_2_ formation, where the {110} surface becomes the most favorable facet followed by the {111} and {100} surfaces. Unlike for pristine surfaces, the formation of H_2_O_2_ on oxygen deficient surfaces does not show any clear trend as *x*_s_(Ce^3+^) increases.

Like other mechanisms, the desorption of O_2_ (R11) and H_2_O_2_ (R14) are unfavorable, and the desorption of O_2_ shows lower positive energies than that of H_2_O_2_, Figure 4. Both pristine and oxygen deficient {111} surfaces, across all *x*_s_(Ce^3+^), show the least positive O_2_ and H_2_O_2_ desorption energies.

At all *x*_s_(Ce^3+^), the O_2_ desorption is less energetically demanding on the pristine {111}, followed by {110} and then {100} surfaces (Figure 4a–c). The desorption of O_2_ on oxygen deficient surfaces does not show a clear trend as *x*_s_(Ce^3+^) increases, but the oxygen deficient {110} surface always displays the highest O_2_ desorption energy (Figure 4d–f).

H_2_O_2_ requires the highest energy to desorb from the pristine {110} surface (*x*_s_(Ce^3+^) = 0, Figure 4a), followed by the {100} and {111} surfaces. However, as *x*_s_(Ce^3+^) increases on the pristine surfaces, the {100} surface requires the highest energy to desorb H_2_O_2_, followed by the {110} and {111} surfaces (Figure 4b,c). On the oxygen deficient surfaces, for *x*_s_(Ce^3+^) = 0.50/0.75, the desorption of H_2_O_2_ is more favorable on the {111}, followed by the {110} and {100} surfaces (Figure 4d,e). However, at the *x*_s_(Ce^3+^) = 1, the oxygen deficient {110} surface requires the highest energy to desorb H_2_O_2_, followed by the {100} and {111} surfaces (Figure 4f).

In general, Figure 4 shows the M7 mechanism would not be ideal for SOD catalytic activity due to the unfavorable H_2_O_2_ formation (R13) step, and the high energies to overcome for the desorption of *O*_2_ (R11) and H_2_O_2_ (R14). Hence, the mechanism M7 would still not be better than M4 and M5 as in the latter mechanisms, the formation of H_2_O_2_ (R4) is generally favorable.

#### 3.1.2 The Catalase (CAT) Activity of Ceria

The catalase activity (CAT) is the conversion of 2 H_2_O_2_ molecules into H_2_O and O_2_ as seen in eq 3

3 $${2H}_{2}O_{2}\to {2H}_{2}O+O_{2}$$

Considering eq 3, we propose a catalytic mechanism of CAT on ceria surfaces, first, without oxygen vacancies, and second, with oxygen-deficient ceria surfaces with oxygen vacancies positioned in the surface sublayer (referred to as ssl oxygen deficient surfaces). Oxygen vacancies can also be positioned on the surface layer, but such models allow for the healing of the vacancy resulting in noncatalytic reaction mechanisms (discussed in Section 3.2.2), which do not allow for the reaction in eq 2. As for the SOD catalytic mechanisms (Section 3.1.1), we also label the 2 mechanisms identified for CAT as MX, where M stands for mechanism and X is a number, as shown in Table 2. All mechanisms are depicted schematically in Figure 5.

**Figure 5:**
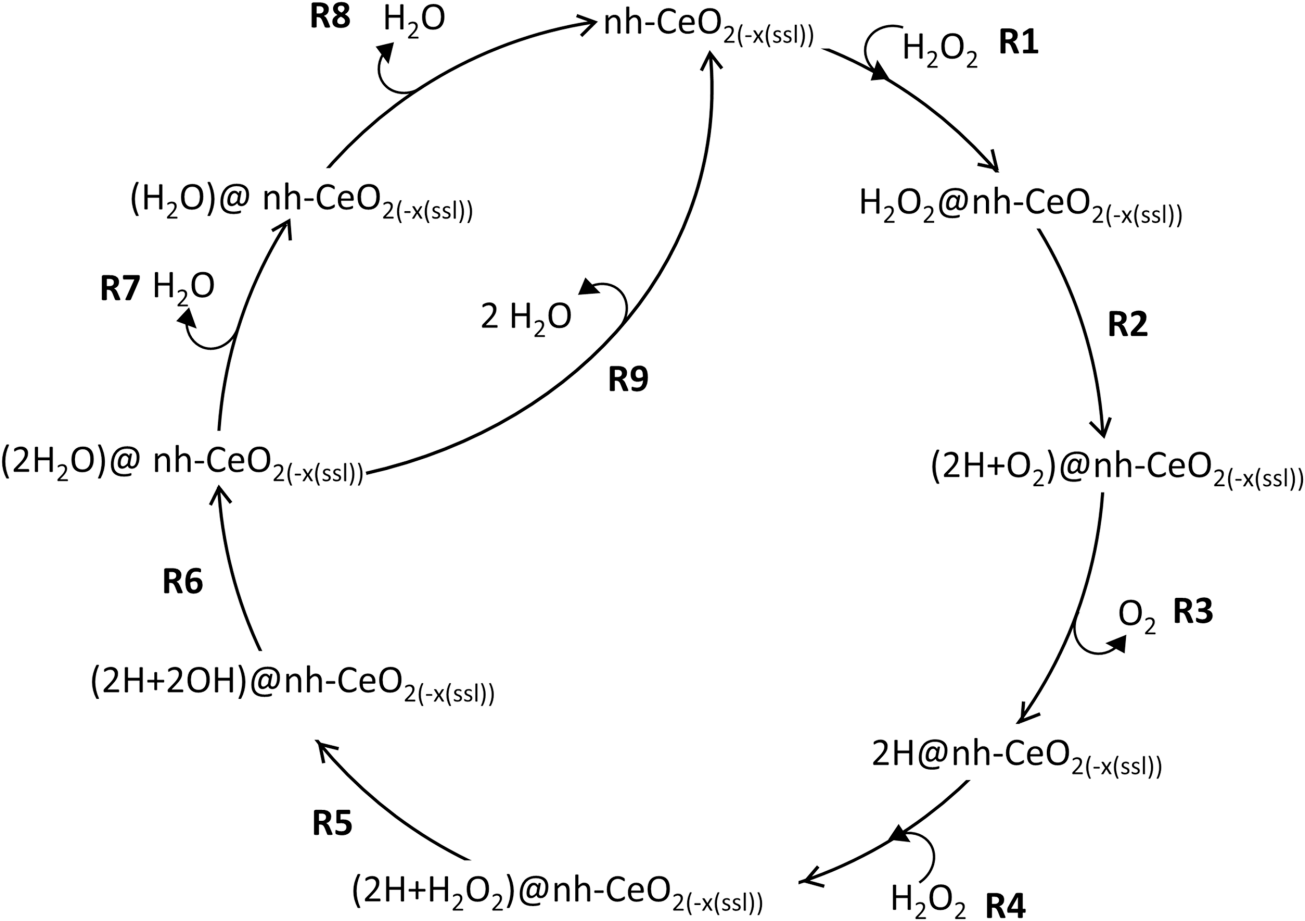
CAT catalytic reaction schemes, applied to the {100}, {110}, and {111} ceria surfaces. The reaction steps are defined by R*y*, where *y* is a number between 1 and 9. The notation *n*h-CeO_2–*x*(ssl)_ represents both pristine (*n*h-CeO_2_) and surface sublayer oxygen deficient (*n*h-CeO_2–*x*(ssl)_) ceria. In sublayer oxygen deficient (*n*h-CeO_2–*x*(ssl)_) ceria, the presence of an oxygen vacancy introduces 2 Ce^3+^ ions into the surface. *n*h represents the number (*n*) of hydroxyl groups at the surface, with *n* varying from 0 to 2, with each introducing 1 Ce^3+^ into the surface.

**Table 2:** Catalytic Reaction Mechanism of CAT According to Figure 5

mechanism	reactions included
M1	R1 + R2 + R3 + R4 + R5 + R6 + R7 + R8
M2	R1 + R2 + R3 + R4 + R5 + R6 + R9

The first adsorbed H_2_O_2_ (R1) is converted into O_2_ (R2) leaving chemisorbed H atoms on the surface of ceria. The O_2_ is then desorbed (R3) before the second H_2_O_2_ molecule is adsorbed (R4) on the surface. The second H_2_O_2_ is then converted into two HO species (R5) before forming 2 H_2_O molecules using H atoms adsorbed on the surface (R6). The two H_2_O molecules can be desorbed consecutively (R7, R8) or simultaneously (R9) as seen in the M1 and M2 mechanisms, respectively.

Similarly to the observations in the SOD activity (Section 3.1.1), where in general, simultaneous desorption is less stable than the consecutive desorption, we see this trend for CAT, where the simultaneous desorption of 2 H_2_O (R9) in M2 requires a higher energy of desorption than the consecutive desorption of water molecules (R7, R8), making M2 a less viable mechanism for CAT activity. Hence, here, we discuss mechanism M1, with its complete energetics shown in Figure 6, while the complete energetics for M2 are shown in Figure S10. The reaction energetics for the oxygen deficient {111} at *x*_s_(Ce^3+^) = 1 for M1 (R5, R6, R7, and R8, Figure 6f), and for M2 (R5, R6, and R9, Figure S10f) are not shown as we could not stabilize the intermediate configurations.

**Figure 6:**
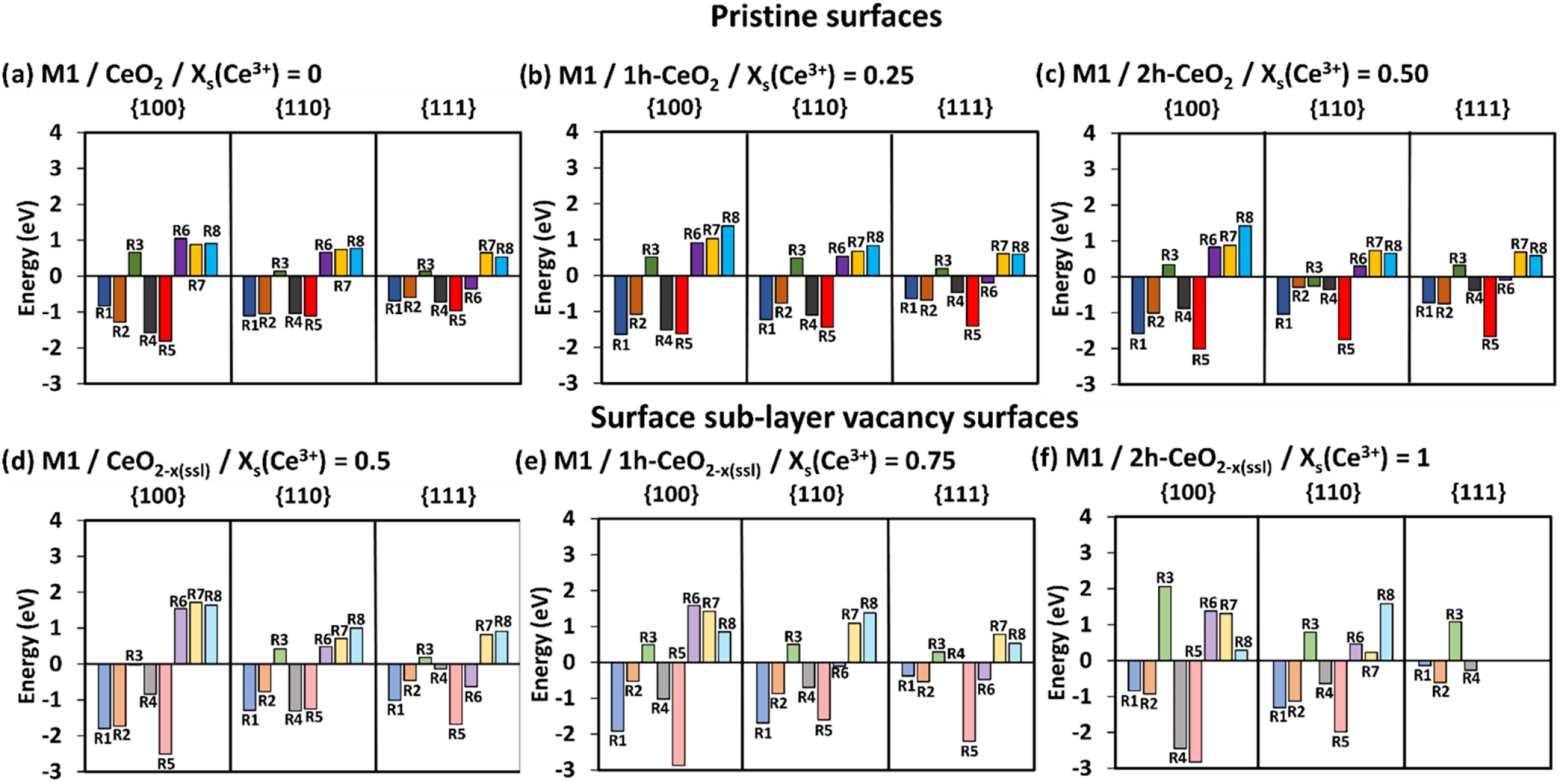
CAT catalytic activity. The complete energetics following mechanisms M1 (a–c) on pristine surfaces and M1 (d–f) on surface sublayer oxygen deficient surfaces at different *x*_s_(Ce^3+^). The darker colored bars are for the pristine surfaces, and the lighter color bars are for the surface sublayer oxygen deficient surfaces. R1/R4 = adsorption of H_2_O_2_, R2 = formation of O_2_, R3 = desorption of O_2_, R5 formation of hydroxyl species. R6 = formation of H_2_O, R7/R8/R9 = desorption of H_2_O.

The adsorption of H_2_O_2_ molecules (R1 and R4) are favorable on both pristine and oxygen deficient surfaces, and R4 is generally less energetically favorable than R1 (Figure 6). There are a few exceptions where R4 is more favorable than R1, including pristine {100} and {111} (R1 {100} = −0.83 eV vs R4 {100} = −1.58 eV, and R1 {111} = −0.69 eV vs R4 {111} = −0.72 eV, Figure 6a), and oxygen deficient {100} and {111} surfaces (R1 {100} = −0.84 eV vs R4 {100} = −2.45 eV, and R1 {111} = −0.14 eV vs R4 {111} = −0.27 eV, Figure 6f). At *x*_s_(Ce^3+^) = 0, R1 is more favorable on pristine {110} surfaces, while R4 is more favorable on pristine {100} surface (Figure 6a). As *x*_s_(Ce^3+^) increases, the adsorption of H_2_O_2_ molecules (both R1 and R4) is more favorable on pristine {100} surfaces, followed by {110} and {111} surfaces (Figure 6a–c), apart from the *x*_s_(Ce^3+^) = 0.50, where R4 {111} = −0.38 eV is slightly more favorable than R4 {110} = −0.36 eV (Figure 6c). On oxygen deficient surfaces, R1 and R4 are more favorable on the {100}, followed by {110} and {111} surfaces for *x*_s_(Ce^3+^) of 0.50 and 0.75 (Figure 6d,e). At *x*_s_(Ce^3+^) = 1 (Figure 6f), R1 is more favorable on the oxygen deficient {110} (−1.31 eV), followed by {100} (−0.84 eV) and {111} (−0.14 eV) surfaces, while R4 follows the order {100} > {110} > {111} with the {100} surface displaying the most favorable adsorption.

The conversion of H_2_O_2_ into O_2_ (R2) is always a favorable process. Figure 6a,b shows that R2 is preferred on the pristine {100}, followed by {110} and {111} surfaces at the *x*_s_(Ce^3+^) of 0 and 0.25. At the highest *x*_s_(Ce^3+^) of 0.50, the pristine {100} is still more favorable, but the {111} and {110} switch stability (R2 {100} = −1.01 eV, R2 {110} = −0.29 eV and R2 {111} = −0.75 eV, Figure 6c). On oxygen deficient surfaces with *x*_s_(Ce^3+^) = 0.50, O_2_ formation is preferred on the {100} surface with the lowest energy of −1.73 eV, followed by {110} and {111} surfaces (Figure 6d). Increasing *x*_s_(Ce^3+^) results in the switch in stability with R2 becoming more favorable on the {110} surface compared to the {100} surface (Figure 6e,f).

To form water molecules from the adsorption of H_2_O_2_ (R4), the conversion of H_2_O_2_ into two HO species (R5) is essential. Figure 6 shows that R5 energies are strongly favorable, and as *x*_s_(Ce^3+^) increases, R5 becomes more favorable on both pristine and oxygen deficient surfaces. The stability of R5 follows the order {100} > {110} > {111} on pristine surfaces (Figure 6a–c), and {100} > {111} > {110} on oxygen deficient surfaces (Figure 6d–f) for all *x*_s_(Ce^3+^) with {100} observed to be the most stable.

The formation of H_2_O (R6) is generally an unfavorable process on the pristine and oxygen deficient {100} and {110} surfaces (except for oxygen deficient {110} surfaces with *x*_s_(Ce^3+^) = 0.75, R6 = −0.11 eV, Figure 6e), but always favorable on pristine and oxygen deficient {111} surfaces (Figure 6). The most favorable H_2_O formation energies are shown by the pristine (−0.36 eV, *x*_s_(Ce^3+^) = 0, Figure 6a) and oxygen deficient (−0.63 eV, *x*_s_(Ce^3+^) = 0.5, Figure 6d) {111} surfaces. The formation of H_2_O is favored on the {111} followed by {110} and {100} surfaces at all *x*_s_(Ce^3+^) for both pristine and oxygen deficient surfaces. Unlike the pristine and oxygen deficient {100} and pristine {110} surfaces where increasing *x*_s_(Ce^3+^) tends to reduce the energy of R6 (i.e., become less positive), the oxygen deficient {110} shows mixed observations. For both pristine and oxygen deficient {111} surfaces, increasing *x*_s_(Ce^3+^) results in less energetically favorable R6 energies as the energies become less negative.

The desorption of O_2_ (R3) and H_2_O (R7, R8) are unfavorable processes, except for the pristine {110} surface with *x*_s_(Ce^3+^) = 0.50 (R3 {110} = −0.26 eV, Figure 6c) and the oxygen deficient {100} surface with *x*_s_(Ce^3+^) = 0.50 (R3 {100} = −0.03 eV, Figure 6d). The desorption of O_2_ generally requires less energy compared to the desorption of H_2_O, except for the oxygen deficient {100} surface with *x*_s_(Ce^3+^) = 1 (Figure 6f).

The desorption of O_2_ is more favorable on the {111}, followed by {110} and {100} pristine surfaces, across all *x*_s_(Ce^3+^). Considering oxygen deficient surfaces, the {111} surface is still the most favorable facet for the O_2_ desorption, except for *x*_s_(Ce^3+^) = 1 where R3 {100} (2.06 eV) > R3 {111} (1.08 eV) > R3 {110} (0.79 eV) (Figure 6f). The increase in *x*_s_(Ce^3+^) on oxygen deficient surfaces results in more positive O_2_ desorption (R3) energies across all three surfaces.

Like for O_2_ desorption, the {111} generally appears to be the most favorable facet to desorb H_2_O (R7, R8) on both pristine and oxygen deficient surfaces compared to the {100} and {110} surfaces across all *x*_s_(Ce^3+^). However, on pristine surfaces the desorption of H_2_O molecules is less favorable on the {100} compared to the {110} surface across all *x*_s_(Ce^3+^). On the oxygen deficient surfaces, the order of stability is less pronounced.

Like the SOD activity, there are similar key points to be considered for a good catalytic CAT activity:

(1) the adsorption of H_2_O_2_ (R1 and R4) should be favorable to allow the first interaction between reactants and ceria
(2) the conversion steps, the O_2_ formation (R2) and the formation of H_2_O (R6), should be as favorable (i.e., associated with a negative energy) as possible
(3) the desorption of O_2_ (R3) and H_2_O (R7, R8) should be spontaneous and associated with negative energy (although we see many of the desorption energies being generally positive).

Hence, the best compromise for the CAT activity would be the pristine surfaces: {100} with *x*_s_(Ce^3+^) = 0.50, {110} with *x*_s_(Ce^3+^) = 0.50, and {111} with *x*_s_(Ce^3+^) = 0. All these compositions show favorable adsorption energies for H_2_O_2_ (R1, R4), as well as the favorable O_2_ formation energies (R2). The {100} pristine surface with *x*_s_(Ce^3+^) = 0.50 has the lowest H_2_O formation energy as well as the best compromise between the O_2_ and H_2_O desorption energies compared to other {100} surfaces. The {110} pristine surface with *x*_s_(Ce^3+^) = 0.50 shows energetically favorable H_2_O formation (R6) and H_2_O desorption energies (R7, R8), as well as a favorable O_2_ desorption energy (R3). The {111} pristine surface with *x*_s_(Ce^3+^) = 0 has a negative H_2_O formation energy (R6) with the lowest O_2_ (R3) and H_2_O (R7, R8) desorption energies.

### 3.2 Noncatalytic Pathways

#### 3.2.1 The Reaction of HOO^•^ Radicals with the Surface-Layer Oxygen Deficient Surfaces of Ceria

Unlike in the SOD catalytic activity, the adsorption of two HOO^•^ radicals on the oxygen deficient surface where the vacancy is positioned on the surface layer (referred to as sl oxygen deficient surfaces) leads to conversion into H_2_O and O_2_ instead of H_2_O_2_ and O_2_. This is due to one of the adsorbed HOO^•^ radicals healing the surface oxygen vacancy to become a hydroxyl group adsorbed on the surface. The healing process hinders the catalytic cycle and thus a noncatalytic mechanism arises. The overall reaction equation is shown in eq 4, where the O_Surf_^*^ represents the oxygen atom of the HOO^•^ radical that heals the surface oxygen vacancy

4 $$2{\mathrm{HOO}}^{•}\to H_{2}O+O_{2}+O_{\mathrm{Surf}}^{*}$$

We label the 4 mechanisms identified in this work as MX, where M stands for mechanism and X is a number, as shown in Table 3. All mechanisms are depicted schematically in Figure 7.

**Figure 7:**
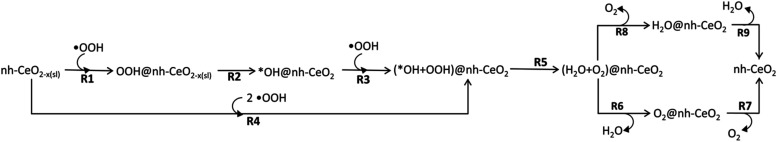
Noncatalytic reaction scheme for the scavenging of HOO^•^ radicals on the {100}, {110}, and {111} oxygen deficient surfaces with the oxygen vacancy positioned on the surface layer (sl). The reaction steps are defined by R*y*, where *y* is a number between 1 and 9. The notation *n*h-CeO_2–*x*(sl)_ represents the surface-layer oxygen deficient (*n*h-CeO_2–*x*(sl)_) ceria, and *n*h-CeO_2_ represents the pristine ceria. In the surface-layer oxygen deficient (*n*h-CeO_2–*x*(sl)_) ceria, the presence of an oxygen vacancy introduces 2 Ce^3+^ ions into the surface. nh represents the number of hydroxyl groups at the surface, with n varying from 0 to 2, with each introducing 1 Ce^3+^ into the surface. The *OH represents the HOO^•^ radical adsorbed onto the surface oxygen vacancy, effectively healing the vacancy.

**Table 3:** Non-Catalytic Reaction Mechanism of SOD According to Figure 7

mechanism	reaction included
M1	R1 + R2 + R3 + R5 + R6 + R7
M2	R1 + R2 + R3 + R5 + R8 + R9
M3	R4 + R5 + R6 + R7
M4	R4 + R5 + R8 + R9

Two HOO^•^ can either adsorb together, where one of the HOO^•^ heals the surface (R1), or consecutively. For consecutive adsorption, the first adsorbed HOO^•^ (R2) heals the oxygen deficient surface (R3) and becomes an HO species before the second HOO^•^ is adsorbed (R4). They are then converted into O_2_ and H_2_O (R5). H_2_O can then desorb first (R6) followed by O_2_ (R7), or *vice versa*, O_2_ first (R8) followed by H_2_O (R9).

Hereafter we discuss the mechanism in groups that have similar pathways and steps.

##### 3.2.1.1 Mechanisms M1 and M2

The mechanisms M1 and M2 share the energies of adsorption of HOO^•^ (R1, R3) as well as the healing process (R2). These are followed by the conversion into H_2_O and O_2_ within a single step (R5). The difference between M1 and M2 lies in the order that the desorption of H_2_O and O_2_ occurs (Figure 7), where M1 sees H_2_O desorption first (R6) followed by O_2_ (R7), and O_2_ desorption occurs first (R8) followed by H_2_O (R9) for M2. As we have studied different surface compositions for each mechanism, including bare and hydroxylated sl oxygen deficient surfaces, we present the complete energetics for the M1 and M2 mechanisms in Figure 8.

**Figure 8:**
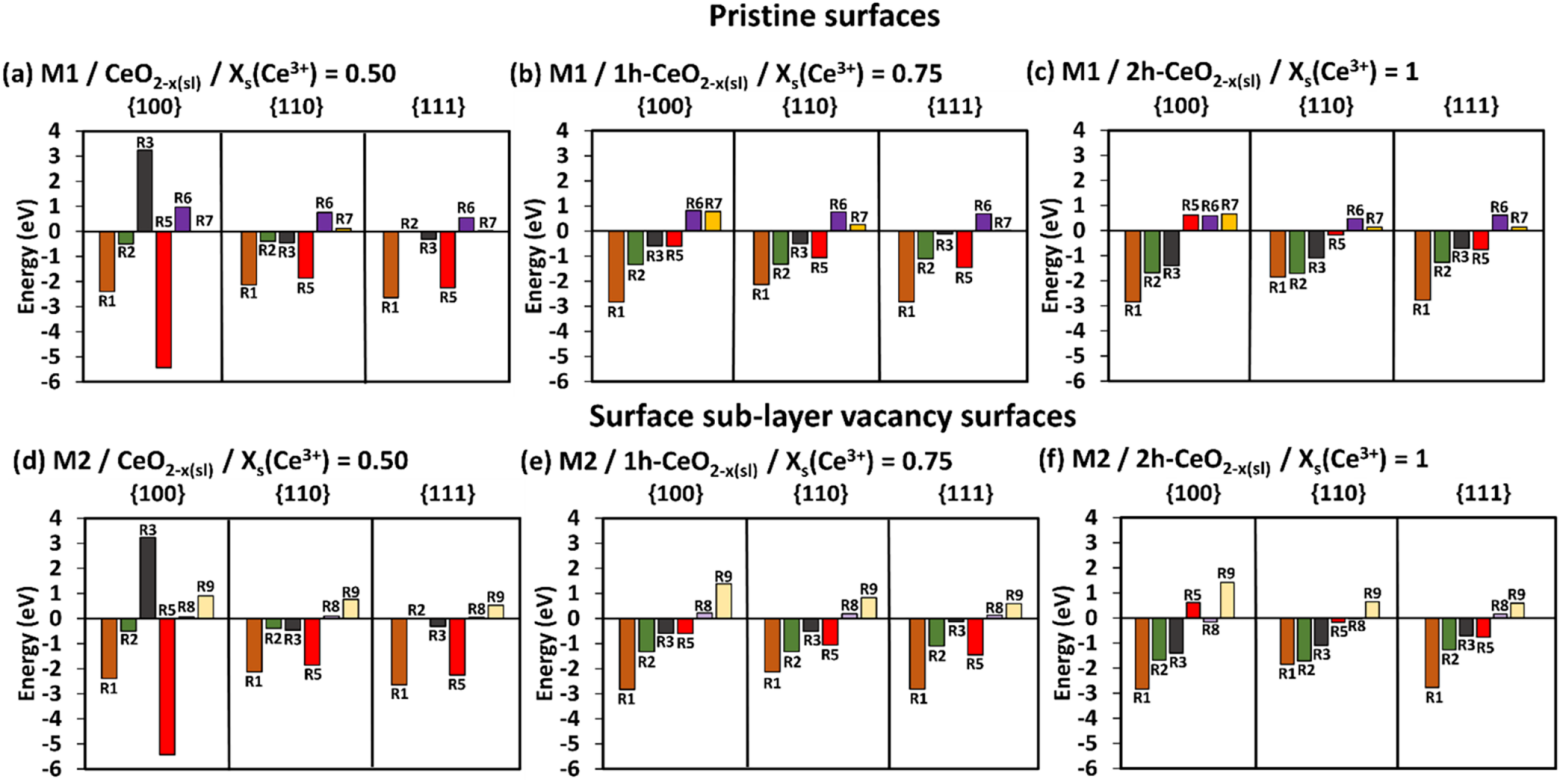
Noncatalytic activity upon HOO^•^ adsorption on the surface-layer oxygen deficient surfaces. The complete energetics following mechanisms M1 (a–c) and M2 (d–f) on the surface-layer oxygen deficient surfaces at different *x*_s_(Ce^3+^). R1/R3 = adsorption of HOO^•^, R2 = healing of the surface vacancy upon HOO^•^ adsorption, R5 = formation of H_2_O and O_2_, R6/R9 = desorption of H_2_O, R7/R8 = desorption of O_2_.

The adsorption of the two HOO^•^ radicals (R1, R3) are favorable, except on the {100} surface at *x*_s_(Ce^3+^) = 0.50, where R3 shows an extremely unfavorable energy (3.24 eV, Figure 8a,d). The adsorption of the first HOO^•^ radical (R1) is favored the most on the {100} followed by the {111} and then the {110} sl oxygen deficient surfaces across all *x*_s_(Ce^3+^). Interestingly, apart from *x*_s_(Ce^3+^) = 0.50, the second HOO^•^ adsorption (R3) follows the energetic order {100} > {110} > {111}, where the adsorption is most favorable on the {100} facet. Similar to the SOD catalytic activity (Section 3.1.1), the second HOO^•^ adsorption is less favorable than that of the first HOO^•^ adsorption across all three surfaces and *x*_s_(Ce^3+^). Both R1 and R3 become more favorable as *x*_s_(Ce^3+^) increases from 0.50 to 1, Figure 8a–f. The different trends observed for the R1 and R3 HOO^•^ adsorption steps are likely to arise from the difference in surface compositions as the first radical heals the surface vacancy of the oxygen deficient surface (R1), while the second adsorbs onto a hydroxylated pristine surface (R3). Although, not directly comparable, it is interesting to note that the adsorption of phosphate on oxygen deficient ceria surfaces follows {100} > {111} > {110}, compared to {100} > {110} > {111} on pristine surfaces.^64^

After being adsorbed onto the surface, the HOO^•^ which is adsorbed first readily heals the oxygen vacancy (R2), and Figure 8 shows R2 energies are all negative across all three surfaces as well as *x*_s_(Ce^3+^). The heat of reduction (i.e., the oxygen vacancy formation energy) of our surfaces in this study using the slab method follows {111} (2.15 eV) > {100} (1.74 eV) > {110} (1.50 eV), and this is comparable with the literature.^57^ The {110} surface has the lowest energy required to create an oxygen vacancy; hence it would be expected to require the lowest energies to be reoxidized again in comparison with {100} and {111}. However, we see that {100} is the most favorable facet for the reoxidization, followed by {110} and then {111} via R2 reaction for all *x*_s_(Ce^3+^). The heat of reduction of the {100} facet is between that of the {111} and the {110}; however, the {100} surface has been reported to have a unique fluid-like property whereas both of the other two surfaces are quite rigid,^64^ hence it is easier for the atoms on the {100} to rearrange themselves for the healing process.

The conversion of the adsorbed HOO^•^ and HO* into H_2_O and O_2_ (R5) is generally a favorable process with the exception of the {100} surface at *x*_s_(Ce^3+^) = 1 (R5 {100} = 0.62 eV, Figure 8c,f). At *x*_s_(Ce^3+^) = 0.50, the formation of H_2_O and O_2_ follows the energetic order {100} (−5.43 eV) > {111} (−2.25 eV) > {110} (−1.86 eV), with the {100} surface seeing an extremely favorable energy (Figure 8a,d). An increase in *x*_s_(Ce^3+^) does not favor H_2_O and O_2_ formation, where we see R5 energies becoming less favorable. The {100} surface is the most affected where the R5 energy becomes unfavorable (i.e., positive) at the *x*_s_(Ce^3+^) = 1 (Figure 8c,f). At high *x*_s_(Ce^3+^), the conversion step R5 is most favorable on the {111}, followed by the {110} and {100} surfaces.

The final step for both the M1 and M2 mechanisms is the desorption of H_2_O (R6, R9) and O_2_ (R7, R8) molecules, which are generally unfavorable. Yet, exceptions exist. The desorption of O_2_ (R8) in the M2 mechanism is favorable on the {100} (R8 = −0.16 eV) and {110} (R8 = −0.04 eV) surfaces at *x*_s_(Ce^3+^) = 1 (Figure 8f). Both M1 and M2 mechanisms have an O_2_ desorption (R7, R8) that is more energetically favorable than the H_2_O desorption (R6, R9).

At *x*_s_(Ce^3+^) = 0.50, the desorption of H_2_O (i.e., M1 R6 and M2 R9) are relatively similar (Figure 8 a,d). However, at higher *x*_s_(Ce^3+^), M2 R9 becomes more positive when compared to M1 R6 (Figure 8b,c,8e,f). At *x*_s_(Ce^3+^) = 0.50–0.75, the desorption of H_2_O is most energetically favorable on the {111}, followed by {110} and {100} surfaces for both the M1 and M2 mechanisms. At *x*_s_(Ce^3+^) = 1, while R9 still expresses the same order of stability on the different surfaces, R6 follows a new trend: {110} (0.48 eV) > {100} (0.59 eV) > {111} (0.61 eV), where the {110} surface is the most favorable surface from which to desorb water (Figure 8c).

The O_2_ desorption energies (M1 R7 and M2 R8) at *x*_s_(Ce^3+^) = 0.50 are relatively low (but still positive) across all three surfaces, especially on both the {100} and {111} surfaces, where the R7 and R8 energies are close to 0 eV (i.e., R7 {100} = 0.01 eV, R7 {111} = 0.03 eV, R8 {100} = 0.07 eV and R8 {111} = 0.04 eV, Figure 8a,d). At higher *x*_s_(Ce^3+^), R7 is more energetically favorable on the {111}, followed by the {110} and the {100} surfaces (Figure 8b,c). However, R8 does not show any clear O_2_ desorption trend at higher *x*_s_(Ce^3+^) (Figure 8e,f). The increase of *x*_s_(Ce^3+^) results in more positive R7 energies on both the {100} and {111} surfaces (Figure 8a,c). R8 becomes more positive on the {111} surface with increasing *x*_s_(Ce^3+^), while giving rise to mixed results on the {100} and {110} surfaces.

Similar to the catalytic SOD and CAT (Section 3.1), the most promising noncatalytic mechanism according to eq 4 would require the energies of adsorption of the adsorbates and of formation of products to be favorable, and the energies of desorption of the products to be less unfavorable. Hence, the best compromises for the noncatalytic activity for removing HOO^•^ radicals following eq 4 are M1 {100} *x*_s_(Ce^3+^) = 0.75, M1 {110} *x*_s_(Ce^3+^) = 0.75, and M2 {111} *x*_s_(Ce^3+^) = 0.75. These choices all share favorable adsorption of HOO^•^ radicals and conversion to H_2_O and O_2_ (R1-R5) as well as a good compromise of low energy penalties for the desorption of O_2_ and H_2_O (R6–R9).

##### 3.2.1.2 Mechanisms M3 and M4

The mechanisms M3 and M4 are similar to the mechanisms M1 and M2, with the difference being that the two HOO^•^ are adsorbed simultaneously (instead of consecutively) healing the surface oxygen vacancy directly (R4). As the M3 and M4 mechanisms share the reaction steps R5 to R9 with the M1 and M2 mechanisms (Section 3.2.1.1), the discussion about the conversion to H_2_O and O_2_ (R5) and the desorption of H_2_O and O_2_ molecules (R6-R9) remains the same. The complete energetics of the M3 and M4 mechanisms are presented in Figure 9.

**Figure 9:**
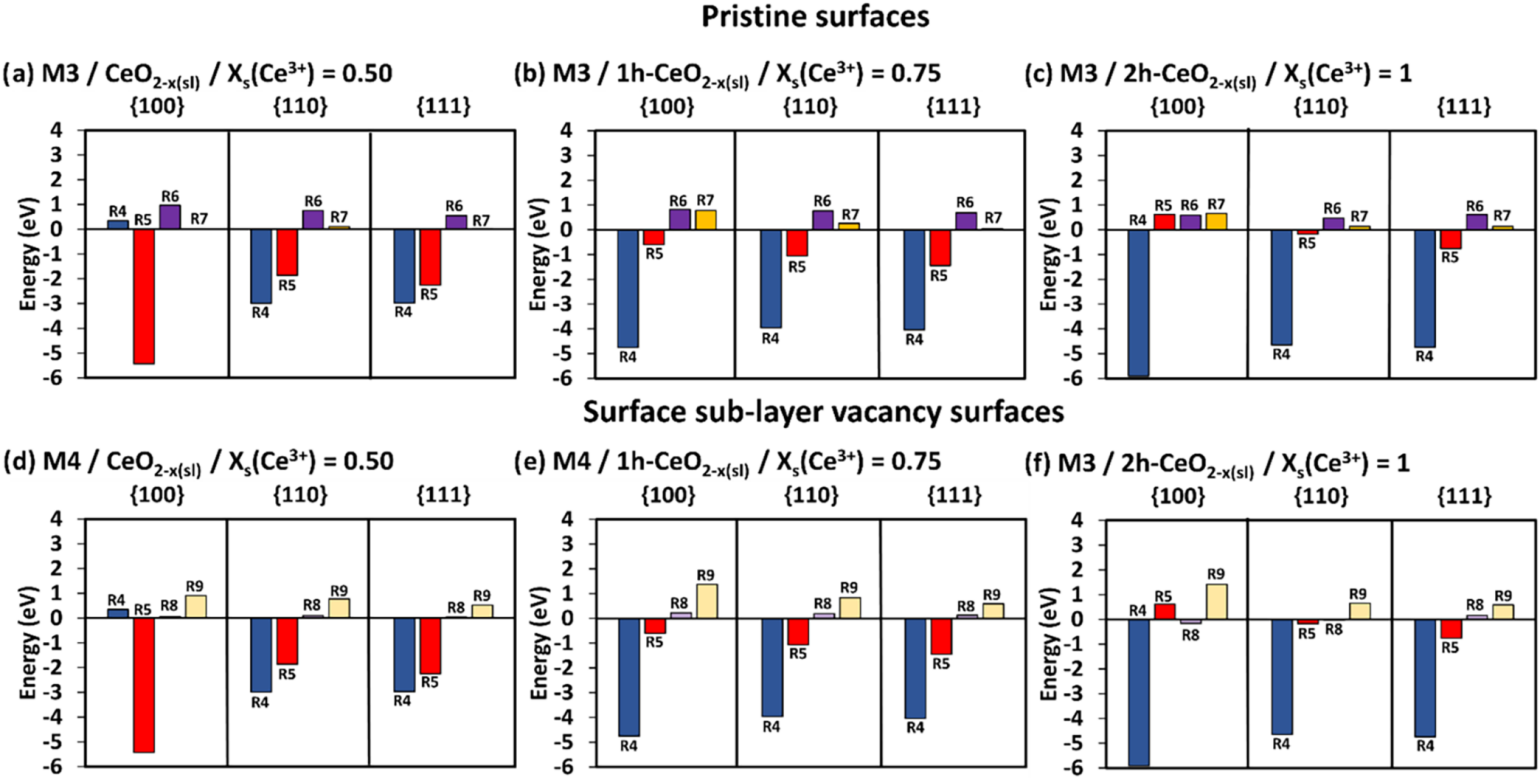
Noncatalytic activity upon HOO^•^ adsorption on the surface-layer oxygen deficient surfaces. The complete energetics following mechanisms M3 (a–c) and M4 (d–f) on the surface-layer oxygen deficient surfaces at different *x*_s_(Ce^3+^). R4 = adsorption of HOO^•^ radicals, R5 = formation of H_2_O and O_2_, R6/R9 = desorption of H_2_O, R7/R8 = desorption of O_2_.

Like the consecutive adsorptions, the simultaneous adsorption of two HOO^•^ radicals (R4) is a favorable process with negative adsorption energies. The only exception is for the {100} surface with *x*_s_(Ce^3+^) = 0.50, where the adsorption energy has a positive value of 0.35 eV, Figure 9a,d. As R4 energies across all *x*_s_(Ce^3+^) are energetically more favorable than those for R1 and R3 in mechanisms M1 and M2 (Figure 8), this suggests that simultaneous adsorption of HOO^•^ radicals onto the ceria surfaces is preferred over the consecutive adsorption of HOO^•^ radicals.

An increase in *x*_s_(Ce^3+^) results in more favorable R4 energies, especially for the {100} surface (i.e., at *x*_s_(Ce^3+^) = 1, R4 {100} = −5.91 eV, Figure 9c,f). At low *x*_s_(Ce^3+^) = 0, the adsorption is preferred on the {110}, then {111} and {100} surfaces, but at higher *x*_s_(Ce^3+^), the adsorption follows the energetic order of {100} > {110} > {111} where the {100} surface is the most favorable facet for the adsorption.

Overall, the best compositions for surfaces to carry out the reaction following eq 4 are M3 *x*_s_(Ce^3+^) = 0.75 for the {100} and {110} surfaces, and M4 *x*_s_(Ce^3+^) = 0.75 for the {111} surface. Furthermore, these will also be better than M1 *x*_s_(Ce^3+^) = 0.75 for the {100} and {110} surfaces and M2 *x*_s_(Ce^3+^) = 0.75 for the {111} surface, as the simultaneous adsorption of HOO^•^ radicals is more favorable than the consecutive adsorption.

#### 3.2.2 The Reaction of H_2_O_2_ Radicals with the Surface-Layer Oxygen Deficient Surfaces of Ceria

Upon the adsorption of H_2_O_2_ on the oxygen deficient surfaces with an oxygen vacancy on the surface layer (R1), H_2_O_2_ is then converted into two HO (hydroxyl) species, one of which heals the surface oxygen vacancy (R2). At the surface, this would correspond to dissociatively adsorbed water on a pristine surface, which can recombine into molecular H_2_O (R3), and desorb (R4), leaving behind a pristine surface. As the oxygen vacancy is healed, this reaction scheme is noncatalytic and follows eq 5. The O_Surf_^*^ represents the oxygen atom which heals the surface oxygen vacancy. The reaction scheme is shown in Figure 10.

5 $$H_{2}O_{2}\to H_{2}O+O_{\mathrm{Surf}}^{*}$$

The adsorbed H_2_O_2_ molecule on the sl oxygen deficient surfaces is converted into two HO species instead of O_2_ as seen in the CAT activity (Section 3.1.2), as the adsorbed H_2_O_2_ is found to preferably adsorb on sl oxygen deficient ceria surfaces as two HO instead of O_2_.^65^ The reaction scheme is not reported as it could not be stabilized on the oxygen deficient {100} surface. The complete reaction energetics on the sl oxygen deficient {110} and {111} surfaces are shown in Figure 11.

**Figure 10:**
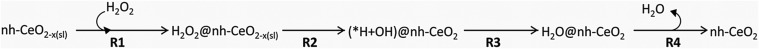
Noncatalytic reaction scheme for the scavenging of H_2_O_2_ on the {100}, {110}, and {111} oxygen deficient surfaces with the oxygen vacancy positioned on the surface layer (sl). The reaction steps are defined by R*y*, where *y* is a number between 1 and 4. The notation *n*h-CeO_2–*x*(sl)_ represents the surface-layer oxygen deficient (*n*h-CeO_2–*x*(sl)_) ceria, and *n*h-CeO_2_ represents the pristine ceria. In the surface-layer oxygen deficient (*n*h-CeO_2–*x*(sl)_) ceria, the presence of an oxygen vacancy introduces 2 Ce^3+^ ions into the surface. nh represents the number of hydroxyl groups at the surface, with *n* varying from 0 to 2, with each introducing 1 Ce^3+^ into the surface. The *H represents the hydroxyl radical adsorbed onto the surface oxygen vacancy, effectively healing the vacancy.

**Figure 11:**
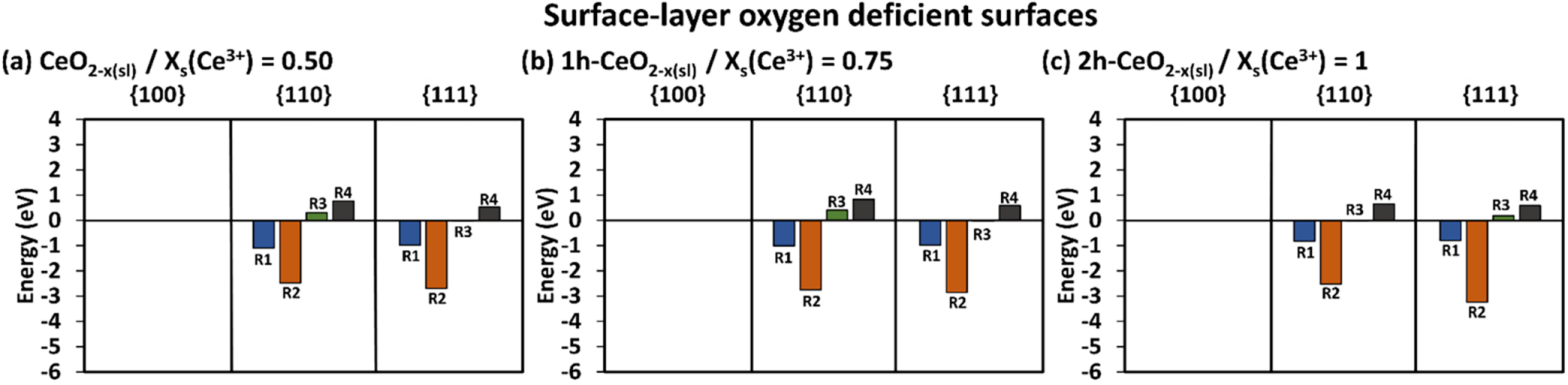
Noncatalytic activity upon H_2_O_2_ adsorption on the surface-layer oxygen deficient surfaces. (a–c) represent the surface-layer oxygen deficient surfaces at different *x*_s_(Ce^3+^). R1 = adsorption of H_2_O_2_, R2 = formation hydroxyl species from the dissociation of H_2_O_2_, where one hydroxyl species heals the surface oxygen vacancy, R3 = formation of H_2_O, R4 = desorption of H_2_O.

The H_2_O_2_ adsorption (R1) is favorable for both the {110} and {111} sl oxygen deficient surfaces with the {110} slightly more preferred than the {111} surface across all *x*_s_(Ce^3+^). An increase in *x*_s_(Ce^3+^) from 0.50 to 0.75 does not have any significant effect on the H_2_O_2_ adsorption energies (Figure 11a,b), however, as the surfaces are fully reduced (i.e., *x*_s_(Ce^3+^) = 1), the adsorption of H_2_O_2_ becomes significantly less favorable on both the {110} and {111} surfaces with R1 {100} = −0.82 eV and R1 {111} = −0.80 eV, Figure 11c. Perhaps, this suggests that Ce^4+^ ions could be better binding sites for the anchoring of H_2_O_2_ molecules compared to Ce^3+^ ions, hence, the absence of Ce^4+^ ions results in a less stable adsorption (Figure 11c).

The conversion of H_2_O_2_ into two HO species and the healing of the oxygen vacancy (R2) is highly favorable with all energies more negative than −2 eV. Across all *x*_s_(Ce^3+^), R2 is more favorable on the {111} than {110} facet. It has been reported that the dissociative adsorption of H_2_O_2_ in terms of two HO on the {111} surface is slightly more favorable than for the {110} surface (−3.66 eV for {111} vs −3.57 eV for {110}),^65^ hence this could contribute to the more favorable R2 energetics observed for {111} compared to the {110} surface.

At *x*_s_(Ce^3+^) of 0.50 and 0.75, the formation of H_2_O is more favorable on the {111} surface with the energies of R3 ∼ 0 eV (but still negative). Conversely, R3 energies are all unfavorable (i.e., positive) on the {110} surface (Figure 11a,b). There is no clear pattern in the formation of H_2_O as *x*_s_(Ce^3+^) increases. At *x*_s_(Ce^3+^) = 1, the formation of water (Figure 11c) is more favorable on the {110} surface (R3 {110} = −0.03 eV) but it is not energetically favorable on the {111} surface (R3 {111} = 0.18 eV).

The desorption of H_2_O (R4) remains an energy penalty with positive desorption energies on all surfaces, slightly less positive on the {111} compared to the {110} facet. An increase in *x*_s_(Ce^3+^) does not appear to significantly influence the desorption, as the R4 energies remain quite consistent across all *x*_s_(Ce^3+^).

Taking into consideration of all the reaction steps (R1–R4) for the noncatalytic conversion of H_2_O_2_, we predict that the {110} *x*_s_(Ce^3+^) = 1 and the {111} *x*_s_(Ce^3+^) = 0.50 would be best to perform this activity (eq 5). Indeed, for these compositions, all energies for the adsorption of H_2_O_2_ (R1), healing of the oxygen vacancy (R2), to the formation of H_2_O (R3) are favorable. As the energies required for the desorption of H_2_O (R4) are quite consistent across all Ce^3+^/Ce^4+^ ratios on both surfaces, thus it would not affect our predictions.

## 4 Discussion

The redox cycle between Ce^3+^ and Ce^4+^ is fundamental for any surface activity, and similarly the oxygen storage capacity of ceria nanoparticles. Catalase (CAT) activity is preferred on ceria at low concentration of Ce^3+^ (i.e., low Ce^3+^/Ce^4+^ ratio), whereas superoxide dismutase (SOD) activity is preferred on ceria at high concentration of Ce^3+^ (i.e., high Ce^3+^/Ce^4+^ ratio).^17,26,28,30,47,61,66^ For example, at pH = 7.2, polycrystalline nanoceria (10–15 nm, expressing mostly the {111} and {100} surfaces) with [Ce^3+^] = 40% showed higher SOD activity than with [Ce^3+^] = 22%.^26^ With regards to CAT, spherical 3–5 nm nanoceria particles, with Ce^3+^/Ce^4+^ = 28.83, showed no detectable activity in comparison with a nanoceria formulation comprised of (irregular) spherical 5–7 nm particles with Ce^3+^/Ce^4+^ = 6.69.^28^ The change in Ce^3+^/Ce^4+^ ratio is shown to shift the SOD to CAT activities (or *vice versa*) of nanoceria. Indeed, a reduction in the Ce^3+^/Ce^4+^ ratio (value not provided) due to phosphate treatment shifted the activity from SOD to CAT.^47^ It has been proposed that the switch between CAT and SOD activities is around [Ce^3+^] = 30–40%.^17,67^ Spherical CeNPs with [Ce^3+^] = 40–58% have displayed mostly SOD activity, while rod-like, cubic and spherical nanoceria with [Ce^3+^] = 26–36% have shown to exhibit CAT activity.^17^ The CAT and SOD efficiency also depends on the shape of the nanoparticles and not only on the [Ce^3+^].^68^ For example, at an equivalent concentration of Ce^3+^, ceria nanorods ([Ce^3+^] = 31.2%) showed 4 times higher SOD activity compared to nanocubes ([Ce^3+^] = 31.8%).^61^ At [Ce^3+^] = 20%, polyhedral (expressing mostly {111}) nanoparticles have been shown to exhibit higher CAT activity (almost double) compared to nanorods (expressing {111} ≈ {100} surfaces) and nanocubes (expressing mostly {100}).^20^ At similar concentrations of Ce^3+^, CAT efficiency has been shown to follow the order of: nanorods ([Ce^3+^] = 50.8%) > nanocubes (41.7%) > octahedra (45.6%), whereas SOD efficiency followed the order of: octahedra > nanocubes > nanorods.^69^ As nanocubes display the {100} surface, nanorods the {110} and {100} surfaces, and octahedra the {111} surface, hence, the different facets also influence the CAT and SOD efficiency of nanoceria, with the {111} surface displaying preferential SOD activity while the {100} and {110} surfaces display preferential CAT activity.

In order to understand the factors controlling the activity of nanoceria, we studied the CAT and SOD activities on the three most stable ceria surfaces (i.e., {100}, {110} and {111}) with different *x*_s_(Ce^3+^) as well as oxygen stoichiometry (i.e., pristine and oxygen deficient surfaces). Our data support the experimental findings by displaying that surface compositions with higher *x*_s_(Ce^3+^) (0.75–1) show a preferential catalytic SOD activity, while surface compositions with lower *x*_s_(Ce^3+^) (0–0.50) show a preferential catalytic CAT activity. Here we attempt to provide some comparison with the available literature.

Our study also highlights the importance of oxygen vacancies and their influence on the reaction pathways. Our data confirm that the adsorption of HOO^•^ and dissociative H_2_O_2_ would heal the surface layer oxygen vacancy and lead to a noncatalytic pathway, while only the pristine and surface sublayer (ssl) oxygen deficient surfaces would express a catalytic pathway for both CAT and SOD activities (i.e., formation of O_2_/H_2_O and H_2_O, respectively, instead of H_2_O_2_/O_2_ and O_2_/H_2_O as seen for the catalytic SOD and CAT activities).^39^ It is clear that noncatalytic pathways would still occur and would gradually reduce the efficiency of catalytic activities over time due to the oxidation of Ce^3+^ active sites.

### 4.1 Catalytic SOD Activity (Figure 1)

Ceria has been shown to have antioxidant activity at low concentrations of hydroxyl radicals.^70^ Our data suggest that HOO^•^ radicals would be quenched (converted into HOO^–^) upon the adsorption on ceria surfaces due to the electron transfer from Ce^3+^ ions. This electron transfer is only possible through a surface adsorbed reactive oxygen species forming a transient surface defect state (TSDS), as demonstrated for some compositions of the {111} ceria surface.^39^ Indeed, the presence of surface defect states (SDSs) alone, such as oxygen vacancies and chemisorbed hydrogen is not sufficient to allow the direct electron transfer, as in the computational findings reported,^39^ both the redox potentials of O_2_/O_2_^•–^ (−0.16 eV) and of O_2_,H^+^/H_2_O_2_ (0.29 eV) would fall between the conduction band and the surface defect states. However, upon the adsorption of reactive oxygen species, as the transient surface defect state (TSDS) forms, it would be in between the two redox potentials allowing for the electron transfer to occur.^39^

We see that the catalytic SOD activity is preferred on the ssl oxygen deficient {100}, {110} and {111} surfaces. This is in line with computational findings on the {111} surface, where the catalytic SOD activity on the {111} pristine surface has a higher energy barrier for the formation of O_2_ and H_2_O_2_ compared to the ssl oxygen deficient surface (1.46 vs 0.89 eV).^39^

We calculated that the difference in energy between the simultaneous adsorption of two HOO^•^ radicals, and an O_2_ and H_2_O_2_ on the ssl oxygen deficient {111} surface has an energy of 0.2 eV (i.e., it is an unfavorable process, mechanism M4 Figure 3d) compared with a previous study that positions this difference at −0.04 eV.^39^ These are calculated as the difference in the adsorption energies of two HOO^•^ radicals (−2.77 vs −2.93 eV^39^) and adsorption of O_2_ and H_2_O_2_ (−2.57 vs −2.97 eV^39^) according to mechanism M4 (Figure 3d), and these differences may be due to the different models used. We used the slab method on thicker slabs with all 5 surface layers allowed to relax during the simulation, whereas the literature^39^ uses the surface method with 1 surface layer fixed and 2 allowed to relax.

The adsorption of one HOO^•^ radical and its conversion to a chemisorbed H and an O_2_ on the {100} pristine surface have been reported with energies of −0.99 and −1.06 eV, respectively.^59^ Our calculated values are not that different at −1.04 and −0.87 eV according to mechanism M7 (Figure 4a). The formation of H_2_O_2_ is an unfavorable process on the pristine {100} surface (R13 = 1.39 eV, M7 Figure 4a, vs 0.67 eV^59^).

Overall, both our pristine (i.e., {100} and {111} surfaces) and ssl oxygen deficient {111} surfaces show comparable adsorption energies toward HOO^•^ radicals, but we see less energetically favorable formation energies for the products compared to the literature.^39,59^

Considering the desorption energies of H_2_O_2_ and O_2_, we see that the desorption of O_2_ is generally less energetically demanding compared to that of H_2_O_2_ on both pristine (R6 = 0.03 eV Figure 2a vs R8 = 0.69 eV Figure 2d) and ssl oxygen deficient (R6 = 0.44 eV Figure 2g vs R8 = 1.01 eV Figure 2j) {111} surfaces. Indeed, for example the adsorption of H_2_O_2_ is more stable than the adsorption of O_2_ on the {111} pristine surface (H_2_O_2_: −0.69 eV and −0.80 eV^39^ vs O_2_: −0.03 and 0.33 eV^58^), which would imply that more energy is required to desorb H_2_O_2_ than O_2_.

The stability of O_2_ adsorption has been reported to be more stable on the bare ssl oxygen deficient {111} surface (i.e., oxygen vacancy in the third layer) compared to the bare pristine {111} surface (−0.06 vs 0.33 eV),^58^ hence the desorption of the O_2_ on the ssl oxygen deficient surfaces would require an energy penalty while it is spontaneous on pristine surfaces. Our energetics complement this observation, where the O_2_ desorption on bare {100}, {110} and {111} ssl oxygen deficient surfaces are 1.8 eV, 0.52 and 0.44 eV (R6, Figure 3d) compared to 0.01 eV, 0.11 and 0.03 eV (R6, Figure 3a) for the pristine {100}, {110} and {111} surfaces, respectively, showing that it is more energetically demanding to desorb O_2_ from oxygen deficient surfaces.

### 4.2 Catalytic CAT Activity (Figure 5)

With regards to the catalytic CAT activity (Section 3.1.2), the adsorption energies of H_2_O_2_ onto the three bare {100}, {110} and {111} pristine surfaces are −0.83, −1.11 and −0.69 eV respectively (Figure 6a), and these are comparable with the H_2_O_2_ adsorption energies reported in the literature for the bare pristine {100} (−0.83 eV^65^), {110} (−1.11 eV^65^), and {111} (−0.69 ^65^ and −0.80 eV^39^) surfaces. On the pristine surfaces, the adsorbed H_2_O_2_ is more preferably adsorbed in a dissociative form of O_2_ and 2 H chemisorbed on the surface, and this behavior has been reported in literature.^39,65^ Considering dissociative adsorption (O_2_ + 2H, R1 + R2 in Figure 6a), we see comparable energies with the literature (i.e., −2.11 vs −2.95 eV^65^ for the {100}, −2.16 vs −1.77 eV^65^ for the {110}, and −1.28 vs −1.29 ^65^ and −1.40 eV^39^ for the {111}). Wang et al. calculated the adsorption of O_2_ and 2H chemisorbed on the ssl oxygen deficient {111} surface to be −1.07 eV and this is slightly less favorable compared to our value (−1.47 eV, R1 + R2 in Figure 6d).

Additionally, our second H_2_O_2_ adsorption (R4 = −0.72 eV, Figure 6a), and the formation of two H_2_O molecules (R4 + R5 + R6 = −2.04 eV, Figure 6a) on the pristine {111} surface are also similar to the literature values of −0.87 eV (for adsorption) and −2.09 eV (for the formation energy of 2 H_2_O).^39^

Our data indicate that pristine surfaces are more beneficial for the promotion of catalytic CAT activity compared to ssl oxygen deficient surfaces. Wang et al. also proved this for the {111} surface indicating that the favorable conversion energy of H_2_O_2_ into O_2_ (−0.60 eV on pristine vs no value reported for the ssl oxygen deficient surface) as the reason for this.^39^ Similarly, we see favorable conversion energies of H_2_O_2_ into O_2_ (R2 = −0.59 eV on pristine Figure 6a, vs R2 = −0.46 eV on ssl oxygen deficient {111} surface, Figure 6d). This would suggest that there will be a higher energy gain in the conversion on the pristine surface. We see also this trend for the {110} (R2 = −1.05 eV on pristine Figure 6a vs R2 = −0.77 eV on ssl oxygen deficient surface Figure 6d) surfaces, but not on the {100} (R2 = −1.28 eV on pristine Figure 6a, vs R2 = −1.73 eV on ssl oxygen deficient surface Figure 6d) surfaces.

Even though Wang et al. did not specify their desorption energies of O_2_ and H_2_O, we suggest that the desorption of the H_2_O and O_2_ could be more of a driving force for an efficient CAT activity. This is due to the departure of the catalytic products that would provide available space for the next adsorbates to adsorb and continue the catalytic cycle. Therefore, the surface configuration that provides the best compromises of less energy-demanding desorption energetics (for both O_2_ and H_2_O) would be more beneficial for such activity. Indeed, our pristine surfaces appear to be generally more beneficial toward the catalytic CAT due to less energetically demanding O_2_ and H_2_O desorption energies across all three pristine surfaces and *x*_s_(Ce^3+^) compared to the ssl oxygen deficient surfaces (Section 3.1.2). Respective O_2_ desorption energies (R3) for the {100}, {110}, and {111} surfaces are 0.66, 0.14, and 0.14 eV for the pristine surfaces (Figure 6a) and −0.03, 0.42, and 0.18 eV for the ssl oxygen deficient surfaces (Figure 6d). In regard to the {100}, {110}, and {111} surfaces, desorption energies of H_2_O molecules (R7, R8) are summarized in Figure 6a,d.

## 5 Conclusions

The pristine and surface sublayer oxygen deficient surfaces can perform catalytic SOD and CAT activities, while the surface layer oxygen deficient surfaces only display a noncatalytic reaction pathway as the surface oxygen vacancy is healed during the reaction process, effectively contributing toward a change in the surface stoichiometry.

We have proposed several mechanisms for both catalytic SOD and CAT activities. In general, we see a complex behavior in terms of the energetics of the different mechanisms. Our modeling displays that the critical points for the mechanism are the adsorption of the adsorbates, the conversion of the adsorbed species, and the desorption of the products. We see that the former two are generally stable, and the latter is unfavorable. For this reason, the desorption of the products would be the rate-determining step of all the catalytic and noncatalytic activities. Depending on the surface fractional coverage of Ce^3+^, we predict that the most beneficial surface compositions for the activities of interest are the oxygen deficient {100} and {110} (*x*_s_(Ce^3+^) = 0.75, M4), and {111} (*x*_s_(Ce^3+^) = 1, M5) surfaces for the catalytic SOD activity, and pristine {100} (*x*_s_(Ce^3+^) = 0.50), {110} (*x*_s_(Ce^3+^) = 0.50), and {111} (*x*_s_(Ce^3+^) = 0) surfaces for the catalytic CAT activity.

While the investigation of the intermediates and transition states provides a necessary approach for testing the feasibility of the proposed reaction steps and indeed, a comparison of the different pathways, we recognize that the determination of activation energies entails further valuable work. This includes computational work which is beyond the scope of this paper on the quantitative determination of reaction rates, via the identification and calculation of the transition state energies between each step, and experimental verification by isolating the more stable reaction intermediates identified here.

## Supplementary Material



## Data Availability

Raw data is available through Mendeley Data at https://doi.org/10.17632/23wkjfjyw3.

## Notes

Catalytical superoxide dismutase (SOD) and catalase (CAT) reaction schematics for the pristine and the surface sublayer oxygen deficient {100}, {110} and {111} ceria surfaces; the noncatalytical reaction schematics when the ^•^OOH radical or the H_2_O_2_ are adsorbed on the surface-layer oxygen deficient {100}, {110} and {111} ceria surfaces (PDF)
