# Supplementary material for: The Buffering Activity of Ceria toward Reactive Oxygen Species: A Density Functional Theory Perspective
Source: J Phys Chem C Nanomater Interfaces. 2025 Jun 18;129(26):11989–2005. doi: 10.1021/acs.jpcc.5c03050 (PMC12235623; doi:10.1021/acs.jpcc.5c03050)
Supplement: Supplementary file 1 [file jp5c03050_si_001.pdf]

Supporting Information

The Buffering Activity of Ceria towards Reactive Oxygen Species: A Density Functional Theory Perspective

Khoa Minh Ta,[1] Craig J. Neal,[2,3] Melanie Coathup,[4] Sudipta Seal,[2,3] Lisa J. Gillie,[1] David J. Cooke,[1] Stephen C. Parker,[5] and Marco Molinari[1,*]

[1]Department of Physical and Life Sciences, School of Applied Sciences, University of Huddersfield, Huddersfield, HD1 3DH, UK

[2]Department of Materials Science and Engineering, Advanced Materials Processing and Analysis Center, Nanoscience and Technology Center, University of Central Florida, Orlando, FL 32816, USA

[3]College of Medicine, University of Central Florida, Orlando, FL 32827, USA

[4]Biionix Cluster, Department of Medicine, College of Medicine, University of Central Florida, Orlando, FL 32827, USA

[5]Department of Chemistry, University of Bath, Claverton Down, Bath BA2 7AY, UK

*Corresponding Author: m.molinari@hud.ac.uk

Figure S1 to S6 are relating to the catalytical superoxide dismutase (SOD) reaction schematics for the pristine and the surface sub-layer oxygen deficient {100}, {110} and {111} ceria surfaces.

Figure S7 to S10 are relating to the catalytical catalase (CAT) reaction schematics for the pristine and the surface sub-layer oxygen deficient {100}, {110} and {111} ceria surfaces.

Figure S11 to S12 are relating to the non-catalytical reaction schematics when •OOH radical adsorbed on the surface-layer oxygen deficient {100}, {110} and {111} ceria surfaces.

Figure S13 to S14 are relating to the non-catalytical reaction schematics when H_2O_2 adsorbed on the surface-layer oxygen deficient {100}, {110} and {111} ceria surfaces.

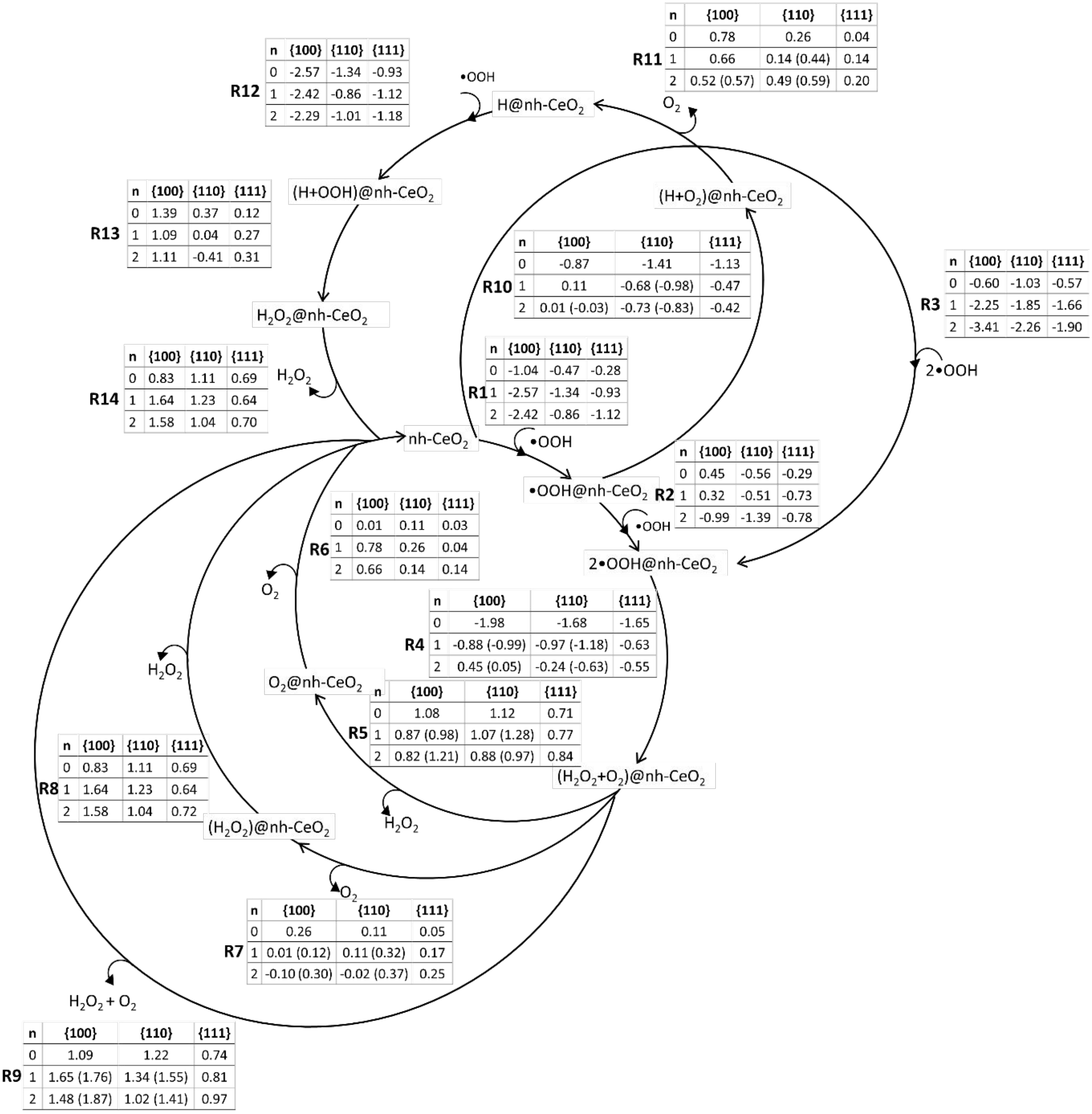

R1

n	{100}	{110}	{111}
0	-1.04	-0.47	-0.28
1	-2.57	-1.34	-0.93
2	-2.42	-0.86	-1.12

R2

n	{100}	{110}	{111}
0	0.45	-0.56	-0.29
1	0.32	-0.51	-0.73
2	-0.99	-1.39	-0.78

R3

n	{100}	{110}	{111}
0	-0.60	-1.03	-0.57
1	-2.25	-1.85	-1.66
2	-3.41	-2.26	-1.90

R4

n	{100}	{110}	{111}
0	-1.98	-1.68	-1.65
1	-0.88 (-0.99)	-0.97 (-1.18)	-0.63
2	0.45 (0.05)	-0.24 (-0.63)	-0.55

R5

n	{100}	{110}	{111}
0	1.08	1.12	0.71
1	0.87 (0.98)	1.07 (1.28)	0.77
2	0.82 (1.21)	0.88 (0.97)	0.84

R6

n	{100}	{110}	{111}
0	0.01	0.11	0.03
1	0.78	0.26	0.04
2	0.66	0.14	0.14

R7

n	{100}	{110}	{111}
0	0.26	0.11	0.05
1	0.01 (0.12)	0.11 (0.32)	0.17
2	-0.10 (0.30)	-0.02 (0.37)	0.25

R8

n	{100}	{110}	{111}
0	0.83	1.11	0.69
1	1.64	1.23	0.64
2	1.58	1.04	0.72

R9

n	{100}	{110}	{111}
0	1.09	1.22	0.74
1	1.65 (1.76)	1.34 (1.55)	0.81
2	1.48 (1.87)	1.02 (1.41)	0.97

R10

n	{100}	{110}	{111}
0	-0.87	-1.41	-1.13
1	0.11	-0.68 (-0.98)	-0.47
2	0.01 (-0.03)	-0.73 (-0.83)	-0.42

R11

n	{100}	{110}	{111}
0	0.78	0.26	0.04
1	0.66	0.14 (0.44)	0.14
2	0.52 (0.57)	0.49 (0.59)	0.20

R12

n	{100}	{110}	{111}
0	-2.57	-1.34	-0.93
1	-2.42	-0.86	-1.12
2	-2.29	-1.01	-1.18

R13

n	{100}	{110}	{111}
0	1.39	0.37	0.12
1	1.09	0.04	0.27
2	1.11	-0.41	0.31

R14

n	{100}	{110}	{111}
0	0.83	1.11	0.69
1	1.64	1.23	0.64
2	1.58	1.04	0.70

Figure S1: The SOD catalytic schematics on the pristine {100}, {110} and {111} surfaces. The nh in nh-CeO_2 represent the amount of H on the ceria surfaces, where it varies from 0 to 2 representing for $x_s(Ce^{3+}) = 0$ to $x_s(Ce^{3+}) = 0.50$. As each chemisorbed H atom introduce 1 Ce^{3+} into the surface and there are 4 Ce^{4+} on our surfaces. The R1-14 notations represent for the order of the reactions. The energies in the bracket represent the reaction energies when the O_2 is not in the triplet form.

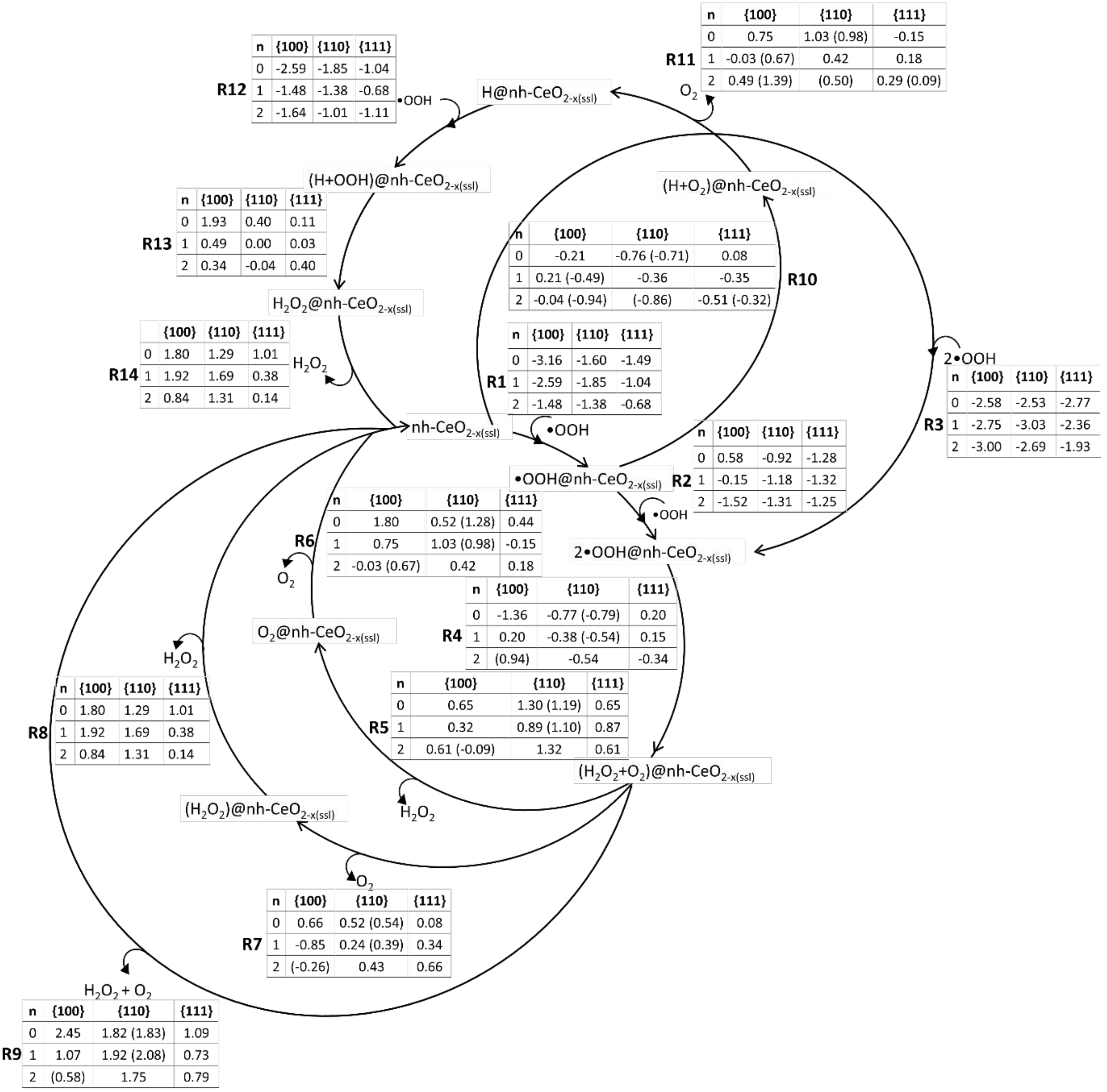

R1

n	{100}	{110}	{111}
0	-3.16	-1.60	-1.49
1	-2.59	-1.85	-1.04
2	-1.48	-1.38	-0.68

R2

n	{100}	{110}	{111}
0	0.58	-0.92	-1.28
1	-0.15	-1.18	-1.32
2	-1.52	-1.31	-1.25

R3

n	{100}	{110}	{111}
0	-2.58	-2.53	-2.77
1	-2.75	-3.03	-2.36
2	-3.00	-2.69	-1.93

R4

n	{100}	{110}	{111}
0	-1.36	-0.77 (-0.79)	0.20
1	0.20	-0.38 (-0.54)	0.15
2	(0.94)	-0.54	-0.34

R5

n	{100}	{110}	{111}
0	0.65	1.30 (1.19)	0.65
1	0.32	0.89 (1.10)	0.87
2	0.61 (-0.09)	1.32	0.61

R6

n	{100}	{110}	{111}
0	1.80	0.52 (1.28)	0.44
1	0.75	1.03 (0.98)	-0.15
2	-0.03 (0.67)	0.42	0.18

R7

n	{100}	{110}	{111}
0	0.66	0.52 (0.54)	0.08
1	-0.85	0.24 (0.39)	0.34
2	(-0.26)	0.43	0.66

R8

n	{100}	{110}	{111}
0	1.80	1.29	1.01
1	1.92	1.69	0.38
2	0.84	1.31	0.14

R9

n	{100}	{110}	{111}
0	2.45	1.82 (1.83)	1.09
1	1.07	1.92 (2.08)	0.73
2	(0.58)	1.75	0.79

R10

n	{100}	{110}	{111}
0	-0.21	-0.76 (-0.71)	0.08
1	0.21 (-0.49)	-0.36	-0.35
2	-0.04 (-0.94)	(-0.86)	-0.51 (-0.32)

R11

n	{100}	{110}	{111}
0	0.75	1.03 (0.98)	-0.15
1	-0.03 (0.67)	0.42	0.18
2	0.49 (1.39)	(0.50)	0.29 (0.09)

R12

n	{100}	{110}	{111}
0	-2.59	-1.85	-1.04
1	-1.48	-1.38	-0.68
2	-1.64	-1.01	-1.11

R13

n	{100}	{110}	{111}
0	1.93	0.40	0.11
1	0.49	0.00	0.03
2	0.34	-0.04	0.40

R14

	{100}	{110}	{111}
0	1.80	1.29	1.01
1	1.92	1.69	0.38
2	0.84	1.31	0.14

Figure S2: The SOD catalytic schematics on the surface sub-layer oxygen deficient {100}, {110} and {111} surfaces. The nh in nh-$CeO_{2-x(ssl)}$ represent the amount of H on the surfaces, where it varies from 0 to 2 representing for $x_s(Ce^{3+}) = 0.50$ to $x_s(Ce^{3+}) = 1$. The presence of oxygen vacancy introduces 2 Ce^{3+} into the ceria surface and each chemisorbed H atom introduce 1 Ce^{3+} into the surface; there are 4 Ce^{4+} on our surfaces. The R1-14 notations represent for the order of the reactions. The energies in the bracket represent the reaction energies when the O_2 is not in the triplet form.

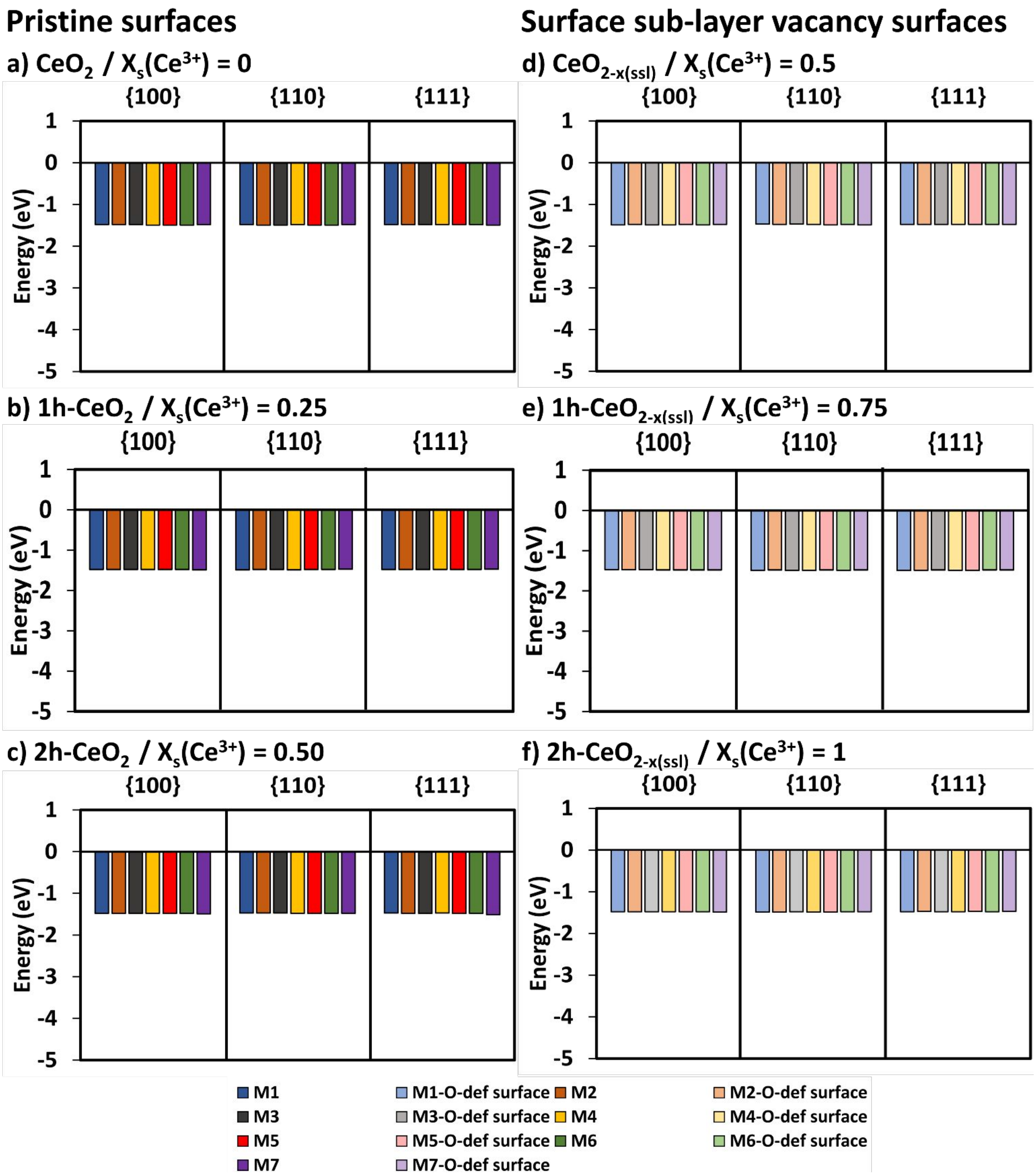

Figure S3: The summary of the total thermal energies of the SOD on the {100}, {110} and {111} nanoceria surfaces following 7 different mechanisms (M1-M7). a-c) represent the energetics on the pristine CeO2 surfaces (darker colour bars). d-f) represent for the energetics on the surface sub-layer oxygen deficient surfaces (lighter colour bars).

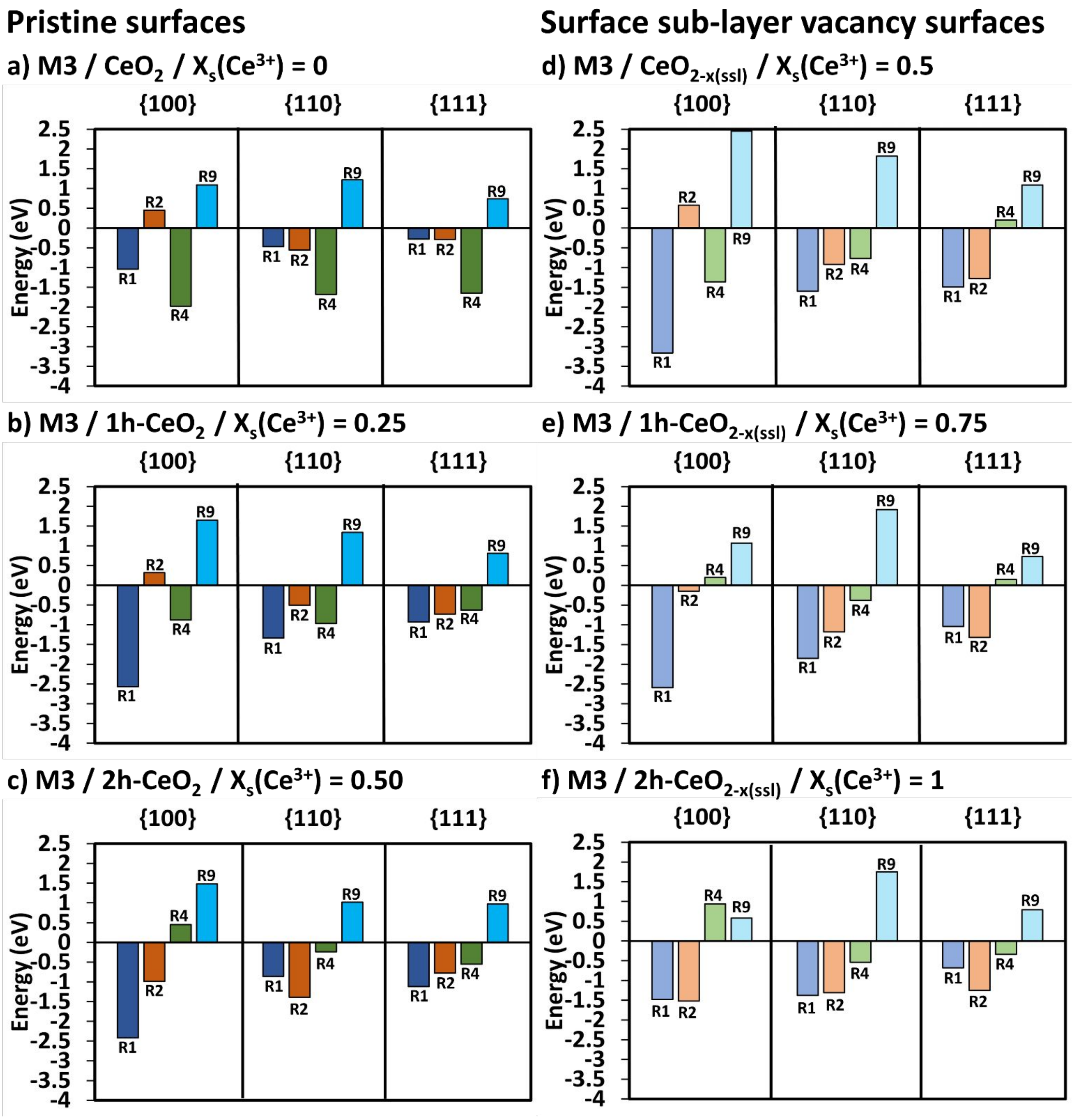

Figure S4: The summary of the energetics following the M3 mechanism of the SOD catalytical scheme on {100}, {110} and {111} ceria surfaces with different surface composition. a-c) are the energetics on the pristine surfaces. d-f) are the energetics on the surface sub-layer oxygen deficient surfaces. R1 = first •OOH adsorption on the surface, R2 = the second •OOH adsorption. R4 = conversion of the two •OOH into H_2O_2 and O_2. R9 = the desorption when both O_2 and H_2O_2 desorbed together.

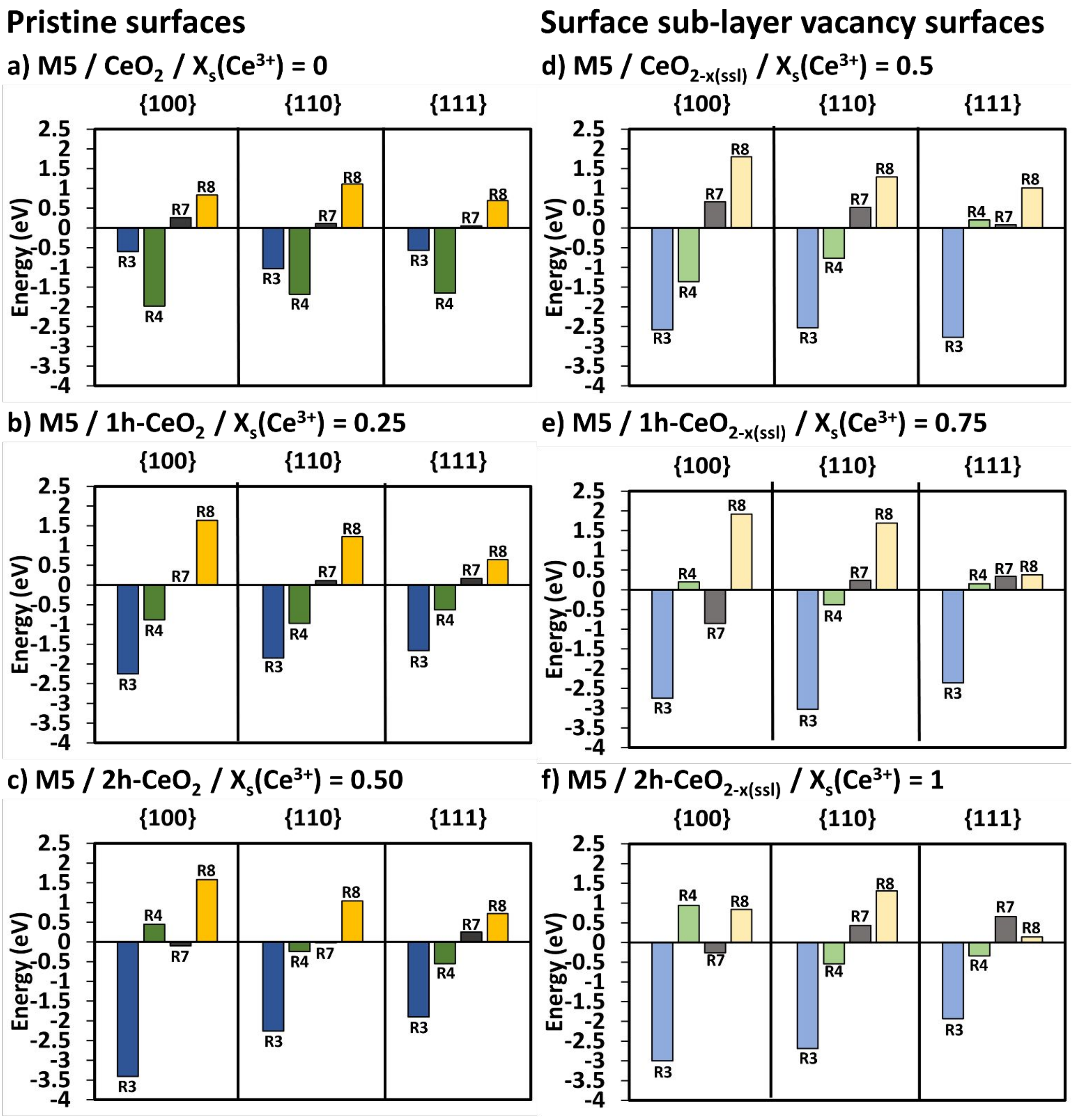

Figure S5: The summary of the energetics following the M5 mechanism of the SOD catalytical scheme on {100}, {110} and {111} ceria surfaces with different surface composition. a-c) are the energetics on the pristine surfaces. d-f) are the energetics on the surface sub-layer oxygen deficient surfaces. R3 = both •OOH adsorbed together on the surfaces. R4 = conversion of the two •OOH into H_2O_2 and O_2. R7 and R8 are the desorption of the O_2 and H_2O_2 correspondingly.

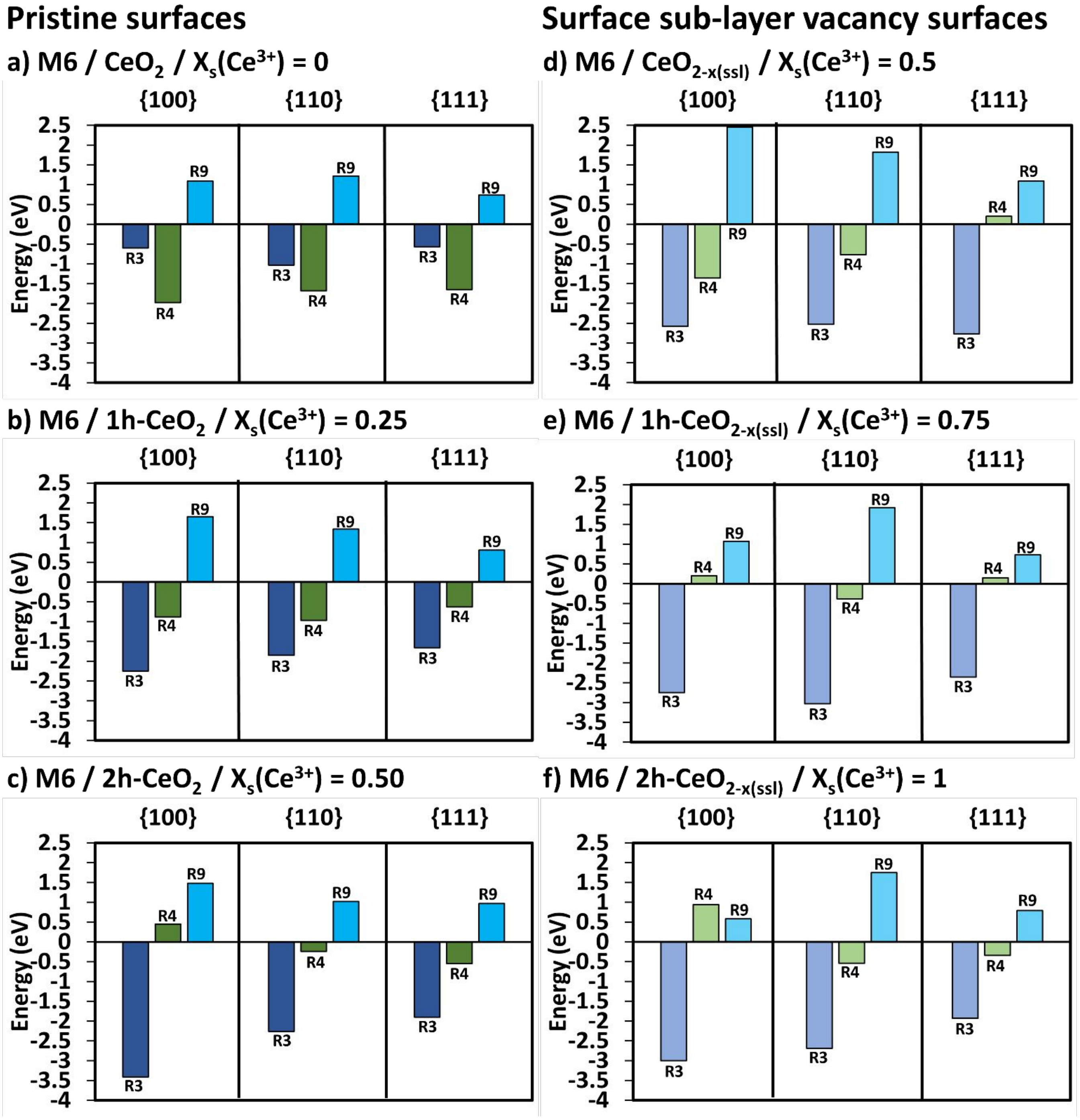

Figure S6: The summary of the energetics following the M6 mechanism of the SOD catalytical scheme on {100}, {110} and {111} ceria surfaces with different surface composition. a-c) are the energetics on the pristine surfaces. d-f) are the energetics on the surface sub-layer oxygen deficient surfaces. R3 = both •OOH adsorbed together on the surfaces. R4 = conversion of the two •OOH into H_2O_2 and O_2. R9 = desorption of the O_2 and H_2O_2.

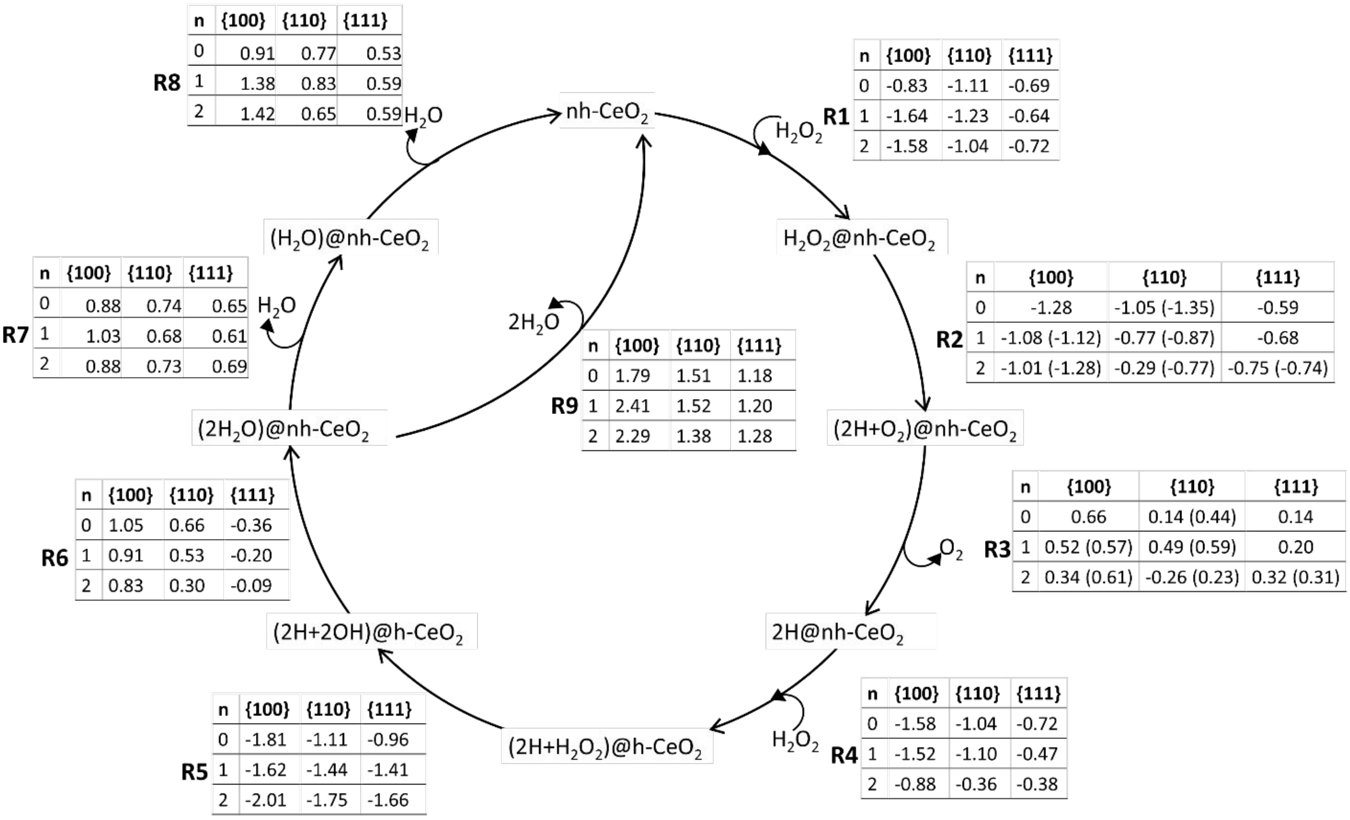

R1

n	{100}	{110}	{111}
0	-0.83	-1.11	-0.69
1	-1.64	-1.23	-0.64
2	-1.58	-1.04	-0.72

R2

n	{100}	{110}	{111}
0	-1.28	-1.05 (-1.35)	-0.59
1	-1.08 (-1.12)	-0.77 (-0.87)	-0.68
2	-1.01 (-1.28)	-0.29 (-0.77)	-0.75 (-0.74)

R3

n	{100}	{110}	{111}
0	0.66	0.14 (0.44)	0.14
1	0.52 (0.57)	0.49 (0.59)	0.20
2	0.34 (0.61)	-0.26 (0.23)	0.32 (0.31)

R4

n	{100}	{110}	{111}
0	-1.58	-1.04	-0.72
1	-1.52	-1.10	-0.47
2	-0.88	-0.36	-0.38

R5

n	{100}	{110}	{111}
0	-1.81	-1.11	-0.96
1	-1.62	-1.44	-1.41
2	-2.01	-1.75	-1.66

R6

n	{100}	{110}	{111}
0	1.05	0.66	-0.36
1	0.91	0.53	-0.20
2	0.83	0.30	-0.09

R7

n	{100}	{110}	{111}
0	0.88	0.74	0.65
1	1.03	0.68	0.61
2	0.88	0.73	0.69

R8

n	{100}	{110}	{111}
0	0.91	0.77	0.53
1	1.38	0.83	0.59
2	1.42	0.65	0.59

R9

n	{100}	{110}	{111}
0	1.79	1.51	1.18
1	2.41	1.52	1.20
2	2.29	1.38	1.28

Figure S7: The CAT catalytic schematics on the pristine {100}, {110} and {111} surfaces. The nh in nh-CeO_2 represent the amount of H on the ceria surfaces, where it varies from 0 to 2 representing for $x_s(Ce^{3+})$ = 0 to $x_s(Ce^{3+})$ = 0.50. As each chemisorbed H atom introduce 1 Ce^{3+} into the surface and there are 4 Ce^{4+} on our surfaces. The R1-9 notations represent for the order of the reactions. The energies in the bracket represent the reaction energies when the O_2 is not in the triplet form.

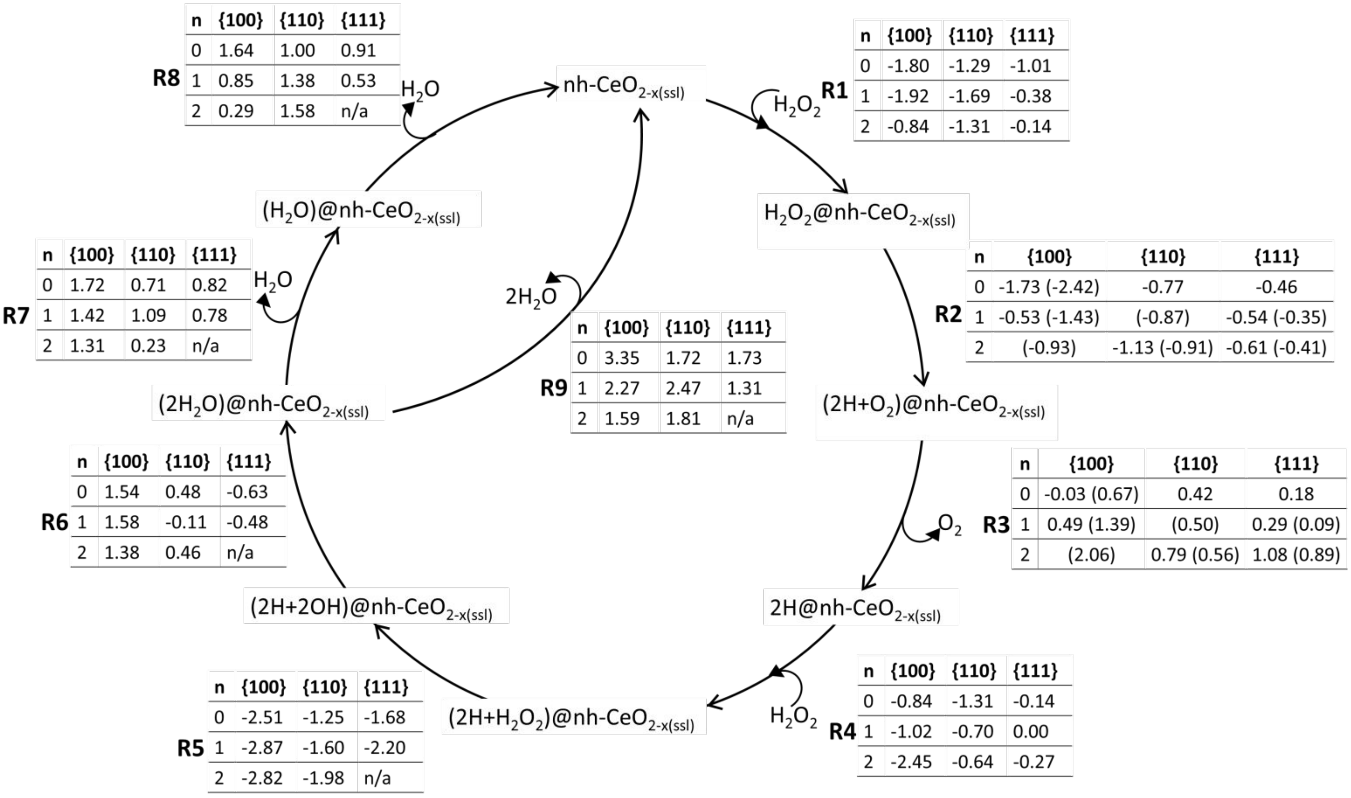

R1

n	{100}	{110}	{111}
0	-1.80	-1.29	-1.01
1	-1.92	-1.69	-0.38
2	-0.84	-1.31	-0.14

R2

n	{100}	{110}	{111}
0	-1.73 (-2.42)	-0.77	-0.46
1	-0.53 (-1.43)	(-0.87)	-0.54 (-0.35)
2	(-0.93)	-1.13 (-0.91)	-0.61 (-0.41)

R3

n	{100}	{110}	{111}
0	-0.03 (0.67)	0.42	0.18
1	0.49 (1.39)	(0.50)	0.29 (0.09)
2	(2.06)	0.79 (0.56)	1.08 (0.89)

R4

n	{100}	{110}	{111}
0	-0.84	-1.31	-0.14
1	-1.02	-0.70	0.00
2	-2.45	-0.64	-0.27

R5

n	{100}	{110}	{111}
0	-2.51	-1.25	-1.68
1	-2.87	-1.60	-2.20
2	-2.82	-1.98	n/a

R6

n	{100}	{110}	{111}
0	1.54	0.48	-0.63
1	1.58	-0.11	-0.48
2	1.38	0.46	n/a

R7

n	{100}	{110}	{111}
0	1.72	0.71	0.82
1	1.42	1.09	0.78
2	1.31	0.23	n/a

R8

n	{100}	{110}	{111}
0	1.64	1.00	0.91
1	0.85	1.38	0.53
2	0.29	1.58	n/a

R9

n	{100}	{110}	{111}
0	3.35	1.72	1.73
1	2.27	2.47	1.31
2	1.59	1.81	n/a

Figure S8: The CAT catalytic schematics on the surface sbu-layer oxygen deficient {100}, {110} and {111} surfaces. The nh in nh-$CeO_{2-x(ssl)}$ represent the amount of H on the surfaces, where it varies from 0 to 2 representing for $x_s(Ce^{3+}) = 0.50$ to $x_s(Ce^{3+}) = 1$. The presence of oxygen vacancy introduces 2 Ce^{3+} into the ceria surface and each chemisorbed H atom introduce 1 Ce^{3+} into the surface; there are 4 Ce^{4+} on our surfaces. The R1-9 notations represent for the order of the reactions. The energies in the bracket represent the reaction energies when the O_2 is not in the triplet form.

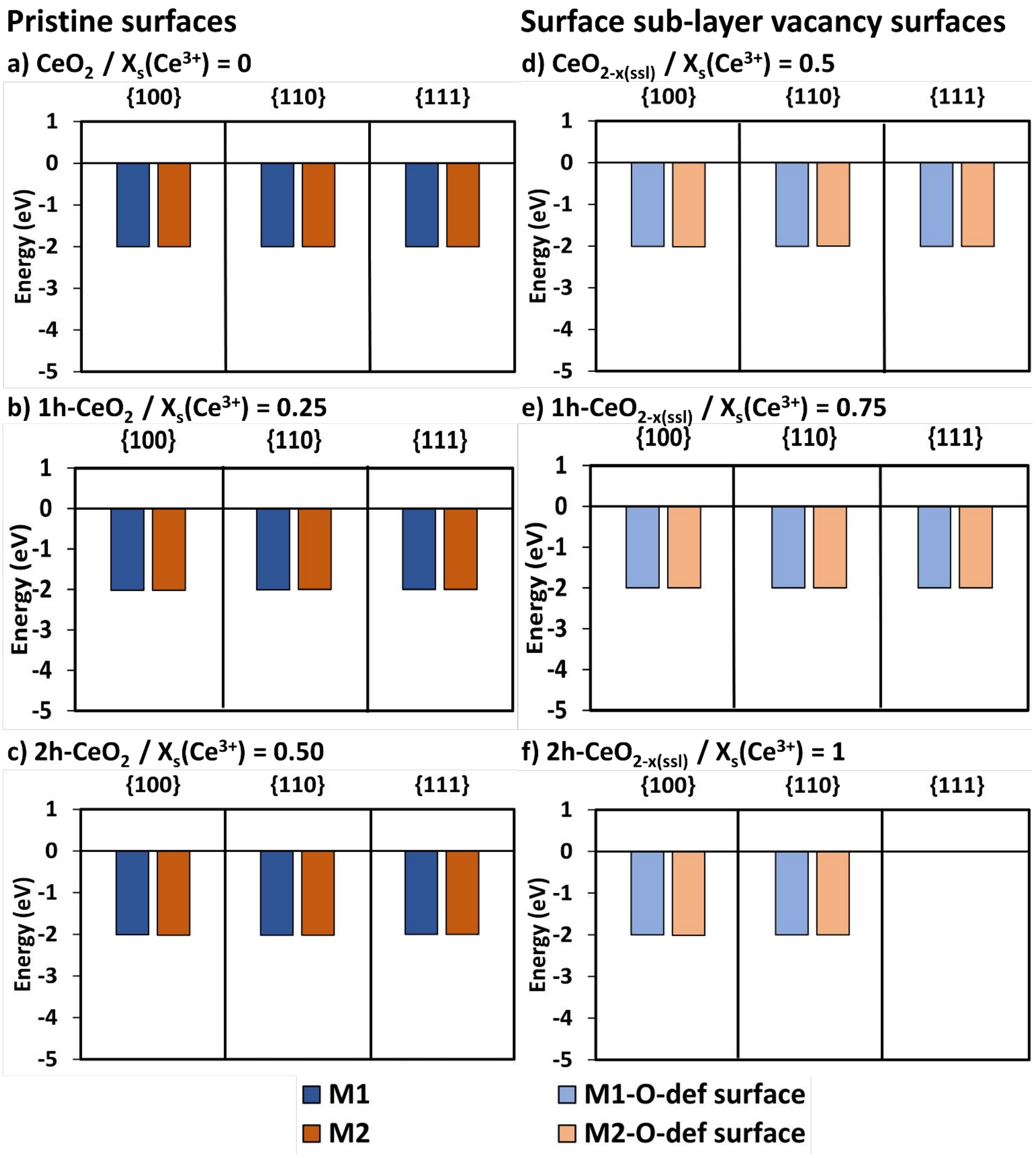

Figure S9: The summary of the total thermal energies of the CAT on the {100}, {110} and {111} nanoceria surfaces following 2 different mechanisms (M1-M2). a-c) represent the energetics on the pristine CeO_2 surfaces (darker colour bars). d-f) represent for the energetics on the surface sub-layer oxygen deficient surfaces (lighter colour bars).

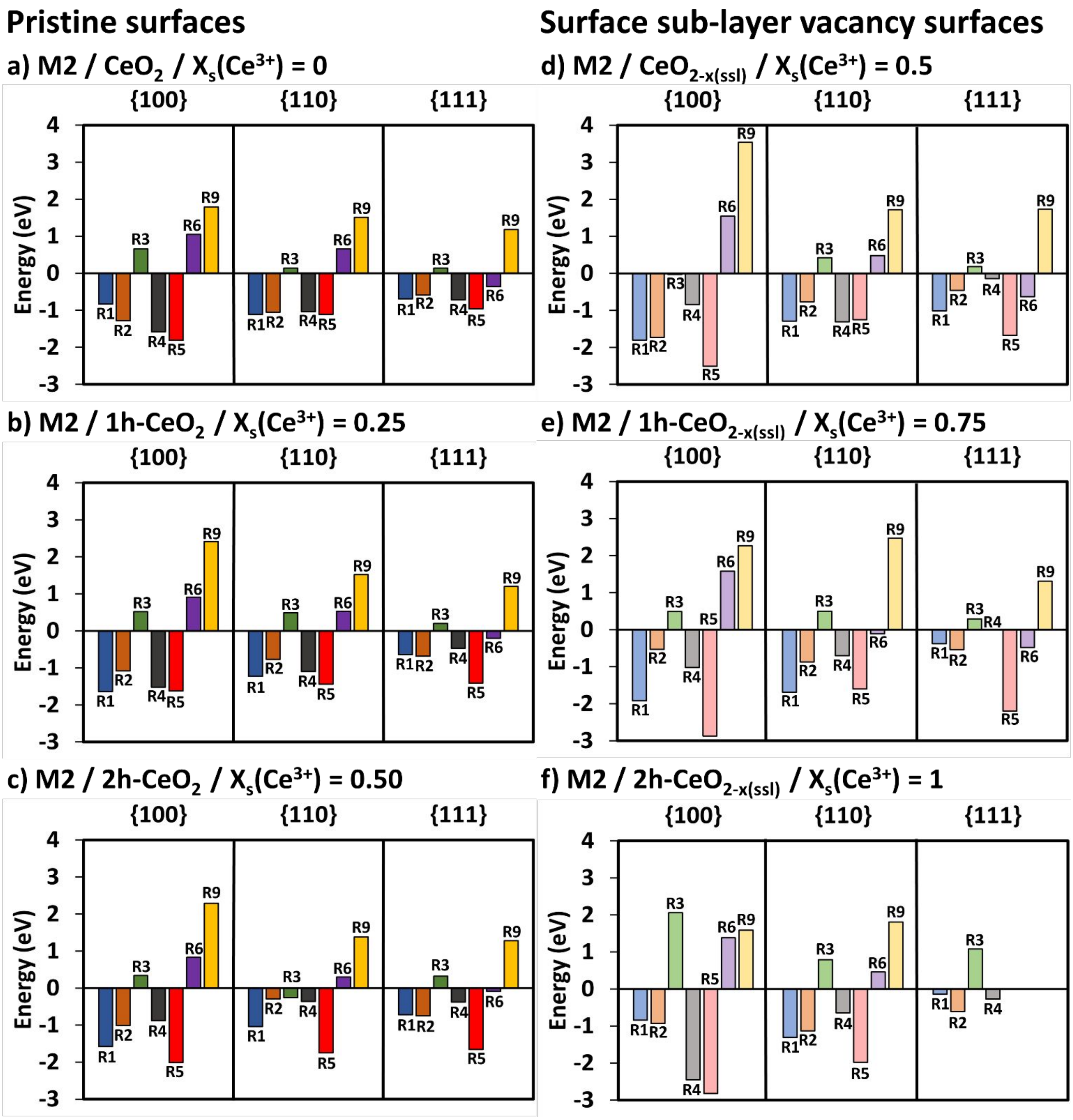

Figure 10: The CAT catalytic activity. The complete energetics following mechanisms M2 (a-c) on pristine surfaces and M2 (d-f) on surface sub-layer oxygen deficient surfaces at different $x_s(Ce^{3+})$. The darker colour bars are for the pristine surfaces, and the lighter colour bars are for the surface sub-layer oxygen deficient surfaces. R1 and R4 are the adsorption of first and second $H2O_2$, R2 = conversion of H_2O_2 into O_2, R3 = desorption of O_2, R5 the conversion of H_2O_2 into 2 OH species. R6 = formation of H_2O, R9 = desorption of both H_2O.

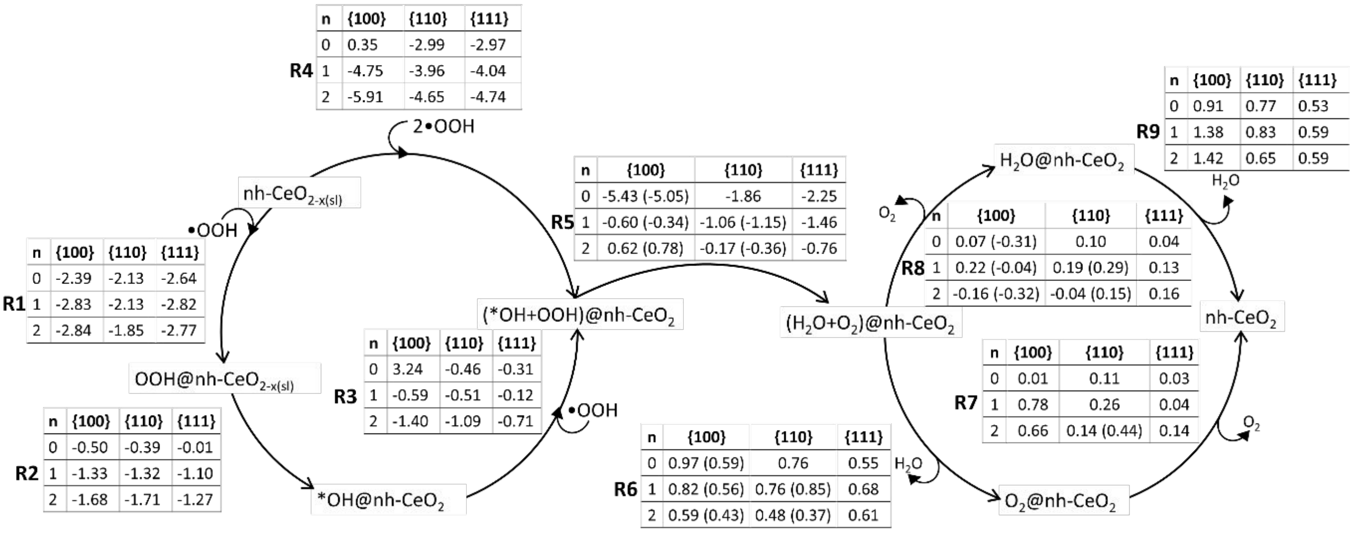

Figure S11: Extra reaction scheme 1. Reaction scheme when two •OOH adsorbed on the surface-layer oxygen deficient {100}, {110} and {111} nanoceria surfaces. This is not a catalytical cycle where the starting surface composition is not retained after the reactions. The nh in nh-$CeO_{2-x(sl)}$ represent the amount of H on the surfaces, where it varies from 0 to 2 representing for $x_s(Ce^{3+})$ = 0.50 to $x_s(Ce^{3+})$ = 1. The presence of oxygen vacancy introduces 2 Ce^{3+} into the ceria surface and each chemisorbed H atom introduce 1 Ce^{3+} into the surface; there are 4 Ce^{4+} on our surfaces. The R1-R9 notations represent for the order of the reactions. The *OH is for the •OOH species where its O atom healed the oxygen vacancy on the ceria surfaces. The energies in the bracket represent the reaction energies when the O_2 is not in the triplet form.

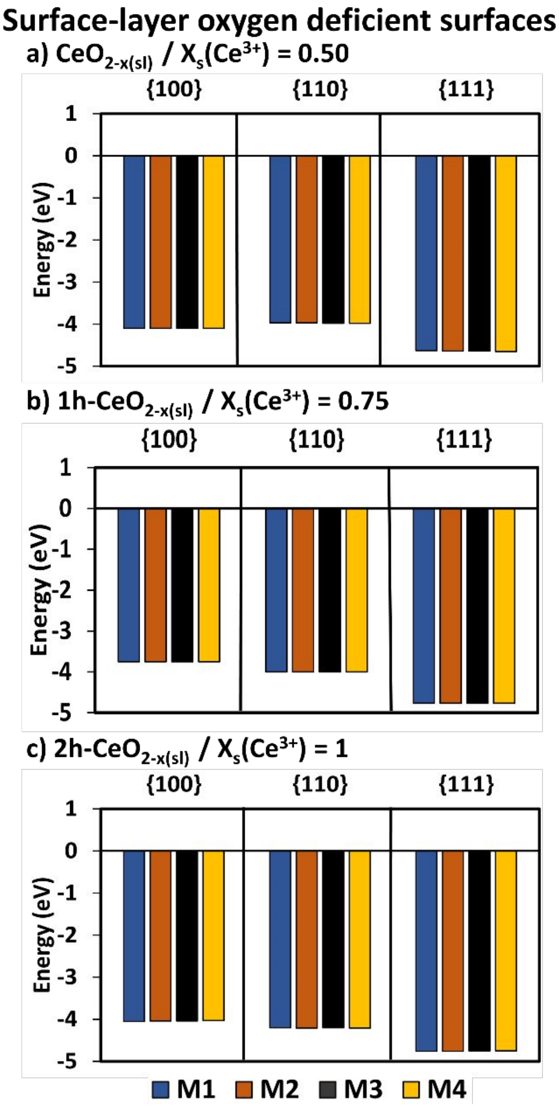

Figure S12: The total energetics for the non-catalytical reaction scheme when two •OOH adsorbed onto the surface-layer oxygen deficient {100}, {110} and {111} surfaces following 4 different mechanisms (M1-M4).

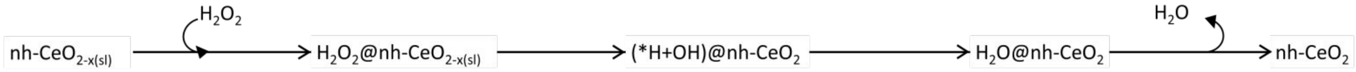

R1

n	{100}	{110}	{111}
0	n/a	-1.09	-0.98
1	n/a	-1.01	-0.98
2	n/a	-0.82	-0.80

R2

n	{100}	{110}	{111}
0	n/a	-2.48	-2.69
1	n/a	-2.75	-2.86
2	n/a	-2.52	-3.24

R3

n	{100}	{110}	{111}
0	n/a	0.30	-0.02
1	n/a	0.41	-0.04
2	n/a	-0.03	0.18

R4

n	{100}	{110}	{111}
0	n/a	0.77	0.53
1	n/a	0.83	0.59
2	n/a	0.65	0.59

Figure S13: The non-catalytical reaction scheme for H_2O_2 adsorption on the surface-layer oxygen deficient surfaces. The nh in nh-$CeO_{2\text{-}x(sl)}$ represent the amount of H on the surfaces, where it varies from 0 to 2 representing for $x_s(Ce^{3+})$ = 0.50 to $x_s(Ce^{3+})$ = 1. The presence of oxygen vacancy introduces 2 Ce^{3+} into the ceria surface and each chemisorbed H atom introduce 1 Ce^{3+} into the surface; there are 4 Ce^{4+} on our surfaces. The R1-R4 notations represent for the order of the reactions. The *H is for the adsorbed OH species where its O atom healed the vacancy on the ceria surfaces.

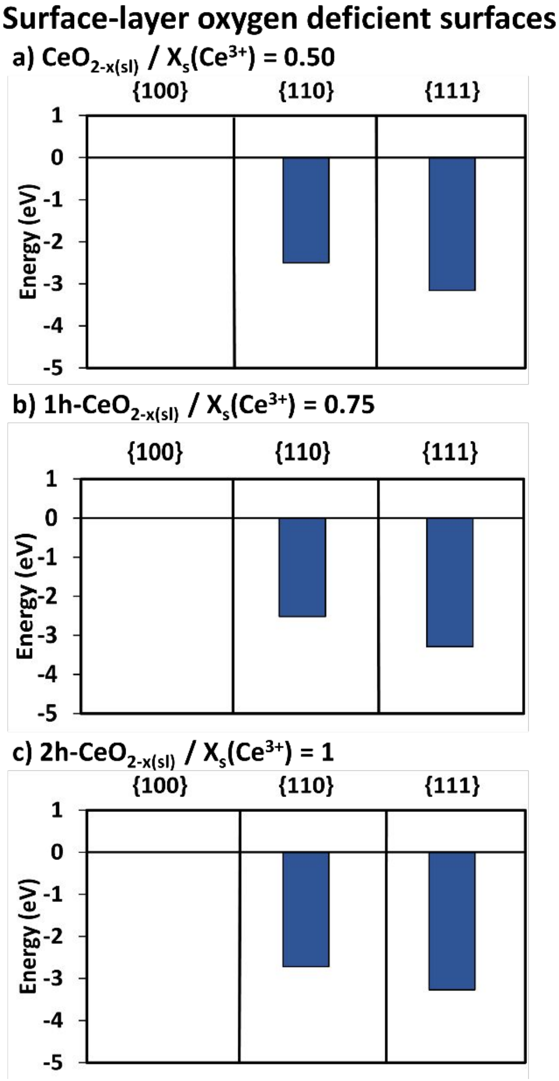

Figure S14: The total energetics for the non-catalytical reaction scheme when H_2O_2 adsorbed on the surface-layer oxygen vacancy {100}, {110} and {111} ceria surfaces.
